# Behavioral, physiological, and genetic drivers of coping in a non-human primate

**DOI:** 10.1016/j.isci.2024.108890

**Published:** 2024-01-12

**Authors:** Debottam Bhattacharjee, Aníta Rut Guðjónsdóttir, Paula Escriche Chova, Esmee Middelburg, Jana Jäckels, Natasja G. de Groot, Bernard Wallner, Jorg J.M. Massen, Lena S. Pflüger

**Affiliations:** 1 Animal Behaviour and Cognition, Department of Biology, Utrecht University, Padualaan 8, 3584 CH Utrecht, the Netherlands; 2 Department of Infectious Diseases and Public Health, Jockey Club College of Veterinary Medicine and Life Sciences, City University of Hong Kong, 31 To Yuen Street, Hong Kong SAR, China; 3 Centre for Animal Health and Welfare, Jockey Club College of Veterinary Medicine and Life Sciences, City University of Hong Kong, 31 To Yuen Street, Hong Kong SAR, China; 4 Department of Behavioral and Cognitive Biology, University of Vienna, Djerassiplatz 1, 1030 Vienna, Austria; 5 Department of Evolutionary Anthropology, University of Vienna, Djerassiplatz 1, 1030 Vienna, Austria; 6 Department of Comparative Genetics & Refinement, Biomedical Primate Research Centre, 2288 GJ Rijswijk, the Netherlands; 7 Austrian Research Center for Primatology, Ossiach 16, 9570 Ossiach, Austria

**Keywords:** Evolutionary biology, Genetics, Zoology

## Abstract

Animals experience stressful situations, from predation to social conflicts, but mostly deal with them successfully. This adaptive mechanism, coping, reduces the adverse effects of stressors, and its failure may result in reduced fitness. Substantial inter-individual variation in coping is observed, yet little is known about how behavioral, physiological and genetic drivers regulate coping holistically and contribute to such variations. We assessed behavioral coping styles (n*=30*), emotional arousal (n*=12*), and personalities (n*=32*) of long-tailed macaques (*Macaca fascicularis*) and also investigated the association of coping with a valine/methionine polymorphism encoded by a critical human stress regulatory gene, catechol-*O*-methyltransferase (COMT) (n*=26*). Personality and the human equivalent *COMT* Val/Met polymorphism were associated with “nonaggression-based” and “aggression-based” coping styles. Compared to nonaggression-based, aggression-based copers maintained higher average facial temperatures, indicating potentially lower emotional arousal, as measured using infrared thermography. These findings demonstrate a complex interplay of various proximate mechanisms governing coping in a non-human primate.

## Highlights

• Long-tailed macaques exhibit “aggression-” and “nonaggression-based” coping styles
• Behavioral coping styles show consistency with emotional arousal
• A human equivalent *COMT* Val/Met polymorphism is present in long-tailed macaques
• Personality and *COMT* Val/Met polymorphism are linked to coping in long-tailed macaques

## Graphical abstract

**Figure:**
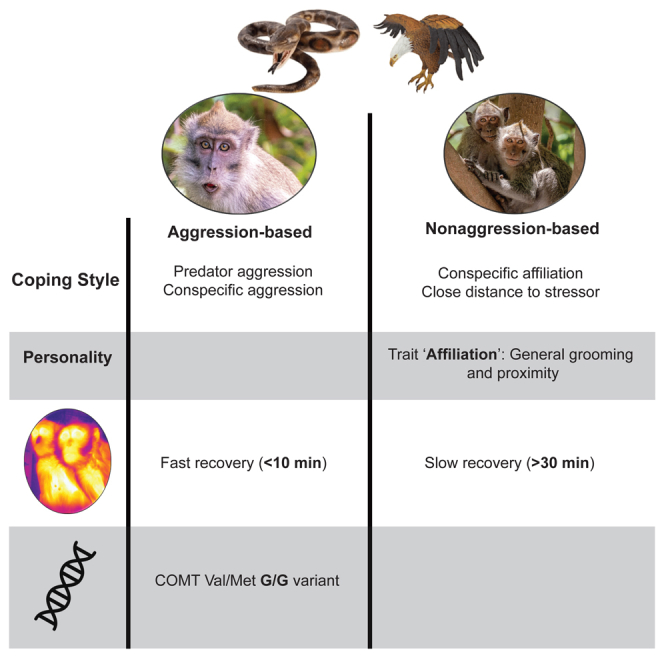

## Introduction

Human and non-human animals are repeatedly exposed to unpredictable and uncontrollable stressors.^1^ The mechanism of coping diminishes the adverse effects of stressors through behavioral and physiological efforts.^2^ Coping has adaptive significance from an evolutionary perspective; failing to cope with stressful situations may result in reduced fitness outcomes, as demonstrated by studies across phylogeny.^3^^,^^4^^,^^5^^,^^6^^,^^7^ Substantial variations are, however, observed in how individuals cope with identical stressors. If those variations among individuals are consistent over time and contexts, they are considered coping styles.^8^ Despite years of theoretical and empirical research, it is still astoundingly challenging to pinpoint the mechanisms linking behavioral traits with stress physiology.^9^^,^^10^ Nevertheless, several theoretical models have been proposed and modified in the last few decades to define the key distinctive properties of coping.^11^ For example, the aggression versus nonaggression coping model highlights that more aggressive individuals show an active response in aversive situations compared to less aggressive individuals who generally aim to reduce the emotional impact of the stressor^12^; and the “problem versus emotion focus” model suggests that while problem-focused coping is directed to encounter and diminish the stressor itself, emotion-focused coping finds alternate ways (such as distancing from the stressor, seeking social support, and so forth) to minimize the harm of distress.^13^ Although the proximal goals of these “alternative” and “subjectively labeled” coping strategies appear different, they are not entirely distinctive in their mechanisms and can complement each other.^14^ Significant advancement has taken place in the scientific research of coping, yet little is known about the complex interplay of behavioral, physiological, and genetic drivers of coping, primarily due to limited existing multidisciplinary studies. Consequently, a holistic understanding of the different proximate mechanisms of coping is obscure. Identifying these mechanisms and quantifying their contributions toward inter-individual variations in coping in non-human animals may thus provide significant insights into its evolution.

In order to explain the behavioral and physiological underpinnings of coping, a 'two-tier' model posits that the responses to stressful situations have two independent dimensions, *coping style* (i.e., a qualitative behavioral dimension: “proactive-reactive continuum,” including traits such as aggression, activity, boldness, and so forth) and *stress reactivity* (i.e., a quantitative dimension: through Hypothalamic-pituitary-adrenal/HPA axis activity).^15^ This model received support from empirical studies conducted mainly on fishes and mammals, such as rodents.^16^^,^^17^^,^^18^ A proactive behavioral coping style indicates an active response to stressors with a higher propensity to take risks and form rigid routines, while a reactive behavioral coping style indicates a passive response through social support-seeking or distancing, and higher behavioral flexibility.^19^ Typically, proactive copers exhibit low inhibition control, high novelty seeking and frequent aggressive and impulsive behaviors^20^^,^^21^; on the contrary, reactive copers do not directly engage with the stressors or show aggressive and impulsive behaviors. At the physiological level, reactive coping is associated with higher HPA axis reactivity than proactive coping.^19^^,^^22^ However, according to the two-tier model, reactive copers can show a low HPA axis- or “emotional” reactivity (cf.^15^^,^^23^) due to the independence of behavioral coping style and HPA axis reactivity. Notably, emotionality in coping has primarily been investigated by measuring stress hormone concentrations (see^24^^,^^25^). Nevertheless, from a neurophysiological perspective, emotional states are associated with the activities of sympathetic and parasympathetic nervous systems, which may increase and decrease blood pressure, resulting in blood flow alterations underneath the skin.^26^^,^^27^ This change in temperature, especially in the face, can thus be used as a reliable alternative for estimating emotional arousal during coping. However, limited information is available on whether such a non-invasive methodological tool can capture or provide information for emotionality in coping.

A key proximate driver of variation in coping styles may be personality. Personality, i.e., consistent inter-individual differences,^20^ are similar in constructs to coping styles and of significant adaptive value.^4^^,^^5^^,^^28^^,^^29^^,^^30^ While both personality and coping styles can be attributed to individual-level differences, the question remains whether coping styles are mere reflections of personalities in stressful contexts. Scientific research in humans predominantly keeps these two concepts fundamentally distinct, and their associations are studied extensively.^31^^,^^32^^,^^33^^,^^34^ Coping, unlike personalities, was found to be less stable over time with adjustments^32^^,^^35^ and had very low heritability.^33^ Furthermore, little to no evidence suggested an overlapping genetic basis of coping styles and personalities.^33^ In contrast to the human literature, non-human animal research often considers coping styles and personalities synonymous. For instance, the 'boldness-shyness' personality dimension^36^ (typically used in birds, fishes, and so forth) is used interchangeably with proactive-reactive coping styles.^19^ Consequently, the concepts of coping (in human and non-human animals) and its (in)dependence on personality need more clarity as variable trait characteristics are subject to selection pressure and are of immense importance.

Conventionally, research on coping has taken an integrated approach of behavior and neurophysiology in non-human animals. As a result, candidate genes of the HPA axis are yet to be significantly explored as potential causal mechanisms. Notably, both coping and personality have some genetic basis, which may work independently or be interlinked through feedback loops.^37^^,^^38^^,^^39^ For example, zebrafish (*Danio rerio*) lines selected for proactive and reactive coping styles show distinct basal neurotranscriptomic states,^40^ and the Swiss sublines of Roman high- and low-avoidance rats selected for good versus poor performance in exploration tasks exhibit divergent stress responses and coping styles.^41^ These findings emphasize the need for studies on key candidate genes regulating stress coupled with behavioral and physiological mechanisms. Such a comprehensive approach is essential for gaining a holistic understanding of coping in non-human animals.

A key candidate gene underlying varying coping styles in humans and potentially other animals is the so-called *COMT* (Catechol-*O*-methyltransferase) gene. COMT is a key enzyme encoded by the *COMT* gene in catecholamine catabolism.^42^ This enzyme is responsible for the inactivation of dopamine, adrenaline and noradrenaline neurotransmitters^43^ and is accountable for more than 60% of dopamine clearance in the brain.^44^^,^^45^ In humans, a single nucleotide polymorphism (SNP) located in the coding region of the *COMT* gene (codon 158, exon 4) has been shown to have functional consequences. At this locus, a transition from guanine (G) to adenosine (A) results in changing the amino acid product from valine (Val) to methionine (Met). The resulting gene products differ significantly in their thermostability and, subsequently, in COMT enzymatic activity. The Val variant is considered more stable and active than the thermolabile Met encoding product.^46^ This functional *COMT* Val^158^Met polymorphism (dbSNP: rs4680) has been studied extensively in humans. Individuals with the G/G (Val) variant are associated with phenotypes, such as higher aggression^47^ and impulsive behavior,^48^ whereas emotional problems^49^ and increased reactivity to stress^50^ are found in individuals with the A/A (Met) variant. In non-human primates (NHP), *COMT* polymorphism is linked with aggression, dominance, and stress.^51^^,^^52^ To our knowledge, however, in the NHP genus *Macaca,* the human equivalent *COMT* Val/Met polymorphism was only identified in Assamese macaques, where a moderate relation between rank and aggression was found.^52^ Although crucial from a mechanistic point of view, no studies to date have investigated the association of *COMT* polymorphism and coping in non-human animals.

The current study uses a multidisciplinary framework to investigate the coping behavior of long-tailed macaques (*Macaca fascicularis*), a group-living NHP species. We conducted ecologically relevant predator exposure experiments repeatedly (see^53^^,^^54^) to assess coping styles. Encounters with predators can be unpredictable and uncontrollable, following the definition of a stressor,^1^ which forces individuals to cope with the situation. An infrared thermal imaging method was employed to detect facial temperature changes during predator exposure, a proxy for emotional arousal.^55^ A drop in nose temperature is typically associated with high emotional arousal in NHP^27^^,^^56^ and thus can provide an estimate of emotionality in coping. We furthermore examined personalities in the long-tailed macaques using a comprehensive multi-method “bottom-up” approach of repetitive behavioral observations and experiments (cf.^53^). Since relatively steep hierarchical structures are found in long-tailed macaque societies,^57^ we also determined the dominance-rank relationships as potential predictors of coping and its variations. Finally, we extracted DNA from blood samples of a subset of subjects to investigate the potential existence of *COMT* polymorphism in the human equivalent *COMT* Val/Met site (exon 4) of long-tailed macaques.

We hypothesized that personality and underlying *COMT* genotype would moderate behavioral coping styles and emotional arousal, explaining individual variations. Due to the nature of the “bottom-up” approach, personality dimensions were not predetermined and thus, informed predictions could not be made. Yet broadly, we predicted that bold- and aggression-related traits would be positively associated with aggression-based coping styles, and social support-seeking behavior would be positively associated with nonaggression-based coping styles (cf.^12^). As high-ranked individuals are often associated with boldness in despotic societies,^58^^,^^59^ we expected them to have aggression-based coping styles compared to their subordinate counterparts. Furthermore, we expected behavioral coping styles and emotional arousal to correlate. Thus, compared to a baseline measure, individuals with aggression-based coping styles are expected to maintain higher average nose temperatures (i.e., potentially low emotional arousal) or recover faster from an initial drop in nose temperature than individuals with nonaggression-based coping styles. Finally, we expected the underlying *COMT* genotypes, if the human-like SNP is present in long-tailed macaques, to be associated with different coping styles in our test subjects. Given the higher emotional resilience attributed to the human G/G genotype (Val carriers),^60^^,^^61^^,^^62^ we would expect long-tailed macaques having the *COMT* G/G genotype (Val) to be less emotionally aroused and to exhibit more aggression-based coping styles compared to macaques carrying the A/A (Met) variant.

## Results

### Behavioral coping styles

Predator models were validated as efficient stressors, as self-directed behaviors were more frequent during predator exposure (0.49 ± 0.33 per minute) than the non-predator baseline (0.27 ± 0.10 per minute) condition (Wilcoxon Signed-Rank test: z = −3.42, r = 0.66, p < 0.001, see Figure S1).

**Figure 1:**
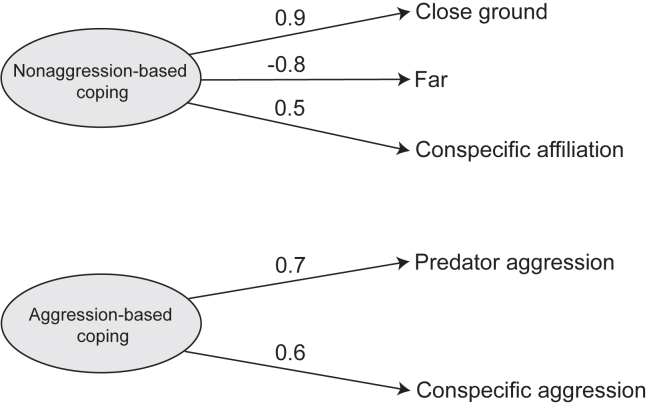
Exploratory factor analysis (EFA) showing two latent factors Latent factors, namely aggression- and nonaggression-based coping, were identified (n = 30). The behavioral variable loadings are indicated by the positive and negative values, see also Table S9.

Considering the behavioral variables we expected to relate to coping (see Table S1), two non-correlating latent factors were extracted using an exploratory factor analysis (EFA). These two factors explained 37.6% and 22.4% of the total observed variance, i.e., 60% cumulatively. The first factor included positively loaded variables of *close ground, conspecific affiliation,* and a negatively loaded variable of *far* (Figure 1). In contrast, the second factor had only two positively loaded variables – *predator aggression* and *conspecific aggression* (Figure 1). Clearly, the second factor had variables relevant to aggression, both toward conspecifics and the stressor; thus, we labeled it as “aggression-based coping” (cf.^12^). The first factor, apart from a close distance, did not indicate any direct engagement with or aggression toward the stressor. Instead, a potential reliance on conspecifics through affiliation was found. We labeled the factor as “nonaggression-based coping” (cf.^12^). In summary, the obtained coping styles indicated direct interactions with conspecifics in the form of aggression or affiliation and direct (with aggression) or indirect (without aggression) engagement with the stressor.

### Coping scores

Individual scores extracted from the factors (i.e., aggression- and nonaggression-based coping) were plotted against each other (see Figure S2). An initial visual inspection was done based on the position of the individuals in the quadrant, where quadrants one, two, three, and four represented mixed, nonaggression-based, low reactivity, and aggression-based coping styles, respectively (see Figure S2).

The aggression-based and nonaggression-based factors had median values of 0.15 and −0.11, respectively. Out of the 30 individuals tested: (i) six belonged to quadrant 1 (mixed coping style); these individuals scored higher than the median scores of both nonaggression- (mean ± standard deviation: (0.30 ± 0.25) and aggression-based (0.80 ± 0.28) coping factors; (ii) Seven individuals belonged to quadrant 2 (nonaggression-based coping style); they scored higher than the median value of the nonaggression-based factor (1.14 ± 0.82) but lower than the aggression-based factor (−0.54 ± 0.58); nonaggression-based factor scores of these individuals were significantly higher than the aggression-based factor scores (Wilcoxon Signed-Rank test: z = 2.41, r = 0.91, p = 0.01); (iii) Seven individuals, who belonged to quadrant 3 (low reactivity category), scored lower than the median values of both the coping factors (aggression-based: −0.88 ± 0.65, nonaggression-based: −0.76 ± 0.76); aggression- and nonaggression-based factor scores were also comparable (Wilcoxon Signed-Rank test: z = 0.72, r = 0.27, p = 0.46); (iv) ten individuals belonged to quadrant 4 (aggression-based coping style), of which nine scored higher than the median score of the aggression-based factor (0.56 ± 0.23); however, all ten of them scored lower than the median value of the nonaggression-based factor (−0.45 ± 0.32). We decided to categorize the particular individual who had an ambiguous value to low reactivity due to the lack of clear evidence of having an aggression-based coping style. Nevertheless, for validation and to check if our downstream analyses were affected by this, we conducted them with and without this individual included as an aggression-based coper. The nine individuals scored higher on the aggression- than nonaggression-based factor (Wilcoxon Signed-Rank test: z = 2.88, r = 0.96, p = 0.003). Interestingly, the six individuals categorized as mixed copers had significantly higher absolute values for the aggression-based factor in comparison to their scores on the nonaggression-based factor (Wilcoxon Signed-Rank test: z = −2.15, r = 0.88, p = 0.03). We combined aggression-based and mixed copers, which further helped us deal with our relatively low sample size. To summarize, except for low reactivity, where specific coping scores could not be assigned (n = 8), individuals belonging to aggression- (n = 15) and nonaggression-based (n = 7) coping styles had specific coping scores from their corresponding factors, i.e., the factor on which they scored (loaded) significantly higher.

At the group level, we found variations in percentages of aggression- and nonaggression-based copers. In Gr.1, ∼36% of the individuals had aggression-based coping styles, ∼53% had nonaggression-based coping styles, and ∼0.9% had low reactivity. Gr. 2 had ∼53% aggression-based copers and, interestingly, no nonaggression-based copers, but ∼46% of the animals had low reactivity. We found 75% and 25% of the monkeys in Gr.3 to have aggression- and nonaggression-based coping styles, respectively.

### Emotional arousal

**Figure 2:**
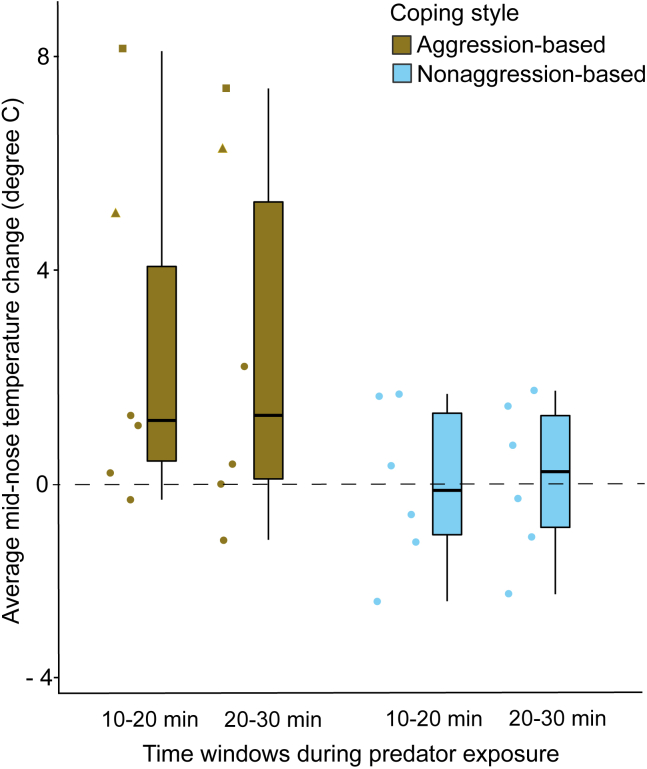
Mid-nose temperature change during predator exposure The boxplot shows the average mid-nose temperature change during predator exposure. Individual data points are represented using solid dots, squares and triangles (n = 12). The squares and triangles indicate individuals with *COMT* Val/Met G/G genotype. Boxes represent interquartile ranges, and whiskers represent the upper and lower limits of the data. The horizontal bars within the boxes represent the median values, see also Figure S3.

According to the best-fitted model (see Table S2) on our thermography data, we found a significant effect of coping style on the nose temperature change (LME: t = −2.490, *η*^*2*^ = 0.45, p = 0.03). In comparison to an initial 2–10 min time window (baseline), individuals with aggression-based coping styles regained and maintained higher average mid-nose temperatures (average change in temperature = 2.54 ± 3.24°C) than those with nonaggression-based coping styles (average change in temperature = 0.03 ± 1.44°C, Figure 2). The temperatures remained consistent for either coping style during the two different time windows (2–10 min vs. 10-20 min - LME: t = 1.815, p = 0.08; 2–10 min vs. 20-30 min - LME: t = 1.889, p = 0.07). Note that the baseline nose temperatures remained comparable between aggression- and nonaggression-based copers (Mann Whitney U Test: z = −1.314, p = 0.17, see Figure S3). We did not find any effect of sex on nose temperature change (LME: t = 1.408, p = 0.19).

A validation test found no significant difference between aggression- and nonaggression-based copers regarding general activity (aggression-based copers: 654.28 ± 253.15 s/h; nonaggression-based copers: 771.98 ± 246.38 s/h; Wilcoxon Rank-Sum test: z = 1.106, p = 0.31), suggesting that the observed nose temperature changes were not due to general movement and foraging, rather an effect of the activities of the sympathetic autonomic nervous system, confirming the emotional arousal we were aiming to measure.

### Personality

Based on the behavioral variables extracted from focal observations and novelty experiments, a principal component analysis (PCA) provided us with three PCs (with eigenvalues >1), cumulatively explaining 78% of the total observed variance (Table 1). We labeled the PCs “Activity-sociability,” “Affiliation,” and “Exploration.” We did not find any effect of age and sex on affiliation; however, independent effects of age and sex were found on activity-sociability and exploration. We found a significant negative effect of age on activity-sociability (GLMM: p = 0.006, see Table S3) and exploration (GLMM: p < 0.001, see Table S4). Males were, furthermore, found to score higher than females in both activity-sociability (female: −0.42 ± 0.66; male: 0.70 ± 1.10; GLMM: p = 0.002, see Table S3) and exploration (female: −0.37 ± 0.73; male: 0.62 ± 1.10; GLMM: p = 0.02, see Table S4) personality traits. Individual scores were obtained for the three traits (see Table S5).

**Table 1:** Output of the principal component analysis

Variables	Activity-Sociability	Affiliation	Exploration	% communalities
Approach	0.93			94.14
Leave	0.92			72.28
Follow	0.85			82.26
Pass by	0.84			87.59
Leave passive	0.83			74.82
Sit	−0.83			75.76
Social play	0.79			87.51
Proximity		0.92		80.88
Groom		0.75		77.41
Handling container			0.97	87.3
Object manipulation			0.66	81.57
Hang			0.54	84.17
% variance	46	17	15	

Variables, factor loadings (>±0.5), attribute communalities, and variance explained by each principal component are provided in the table.

### Personality, dominance hierarchy, and behavioral coping styles

We did not find any effect of the three personality traits, age and sex, on aggression-based coping (see Table S6). Upon the inclusion of the individual with unclear coping style (see section - coping scores; data point highlighted by the solid red dot in Figure S2) as aggression-based coper, our results did not change (null vs. full model comparison: X^2^ = 8.075, p = 0.152). Therefore, we reported and retained the first model results as the main outcome.

**Figure 3:**
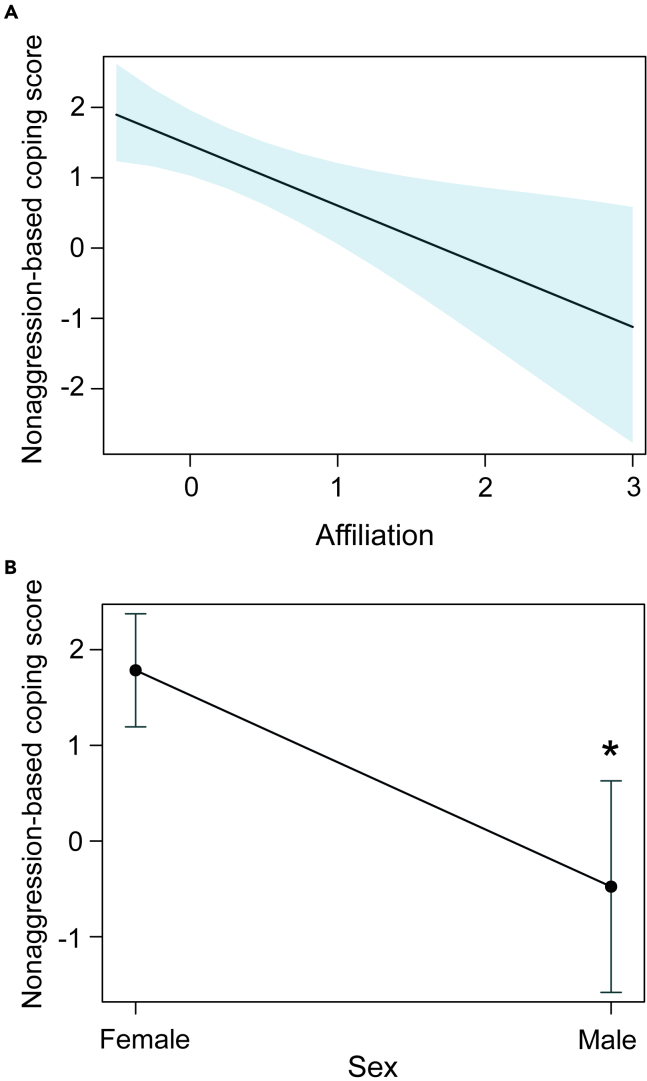
Effect of personality trait affiliation and sex on nonaggression-based coping (A) The effect plot shows an inverse relationship between nonaggression-based coping scores and personality trait affiliation (LME: t = −6.00, *η*^*2*^ = 0.95, 95% CI = 0.32, 1, p = 0.02, n = 7). The shaded area in the figure shows the 95% confidence interval. (B) Females scored higher than males (LME: t = −6.81, *η*^*2*^ = 0.96, 95% CI = 0.42, 1, p = 0.02, n = 7). Data are represented as mean +/− standard error in the figure, see also Table S7.

According to the best-fitted model (see Table S7), we found an effect of the personality trait affiliation (LME: t = −6.00, *η*^*2*^ = 0.95, 95% CI = 0.32, 1, p = 0.02, Figure 3A) and of sex (LME: t = −6.81, *η*^*2*^ = 0.96, 95% CI = 0.42, 1, p = 0.02, Figure 3B) on nonaggression-based coping. An inverse association between affiliation and nonaggression-based coping was found, while females (n = 5; 1.41 ± 0.92) had higher nonaggression-based coping scores than males (n = 2; 0.45 ± 0.21). We did not find any effect of the personality trait exploration on nonaggression-based coping (LME: t = 2.12, p = 0.16) but found a non-significant negative trend of age (LME: t = −3.94, p = 0.05). Note that the non-significant activity-sociability fixed effect was dropped from the best-fitted model during the model selection process.

No correlation was found between aggression-based coping and dominance rank (Spearman’s Rank Correlation: rho = 0.02, p = 0.95). We also found no correlation between nonaggression-based coping and dominance rank (Spearman’s Rank Correlation: rho = −0.39, p = 0.38).

### Catechol-*O*-methyltransferase valine/methionine polymorphism and behavioral coping styles

Sequencing results revealed two alleles equivalent to the human *COMT* Val^158^Met encoding polymorphism in long-tailed macaques. Out of the 26 individuals genotyped, 16 were homozygous A/A (Met encoding allele), six were heterozygous (A/G), and four were homozygous G/G (Val encoding allele). Accordingly, the targeted polymorphism was detected at 61.53%, 23% and 15.38% frequencies, respectively (allele frequencies: A = 0.73; G = 0.27).

**Figure 4:**
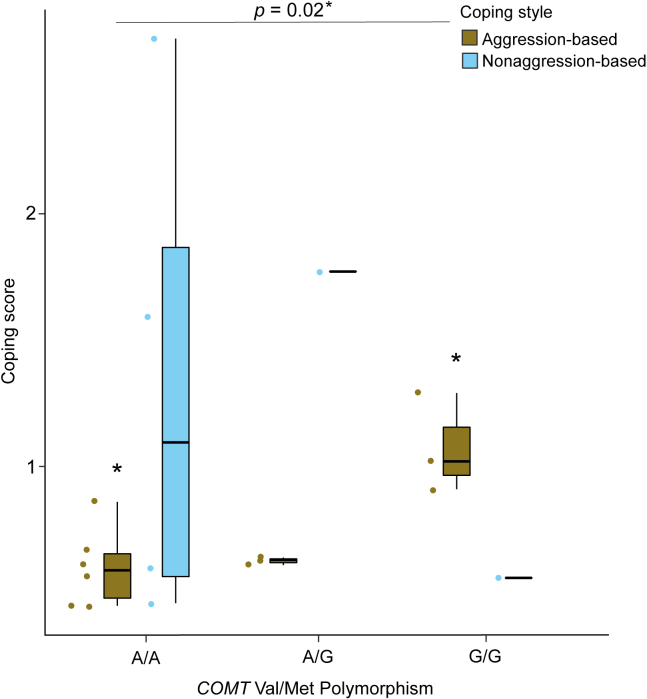
*COMT* Val/Met encoding polymorphism and behavioral coping styles The boxplot shows the two coping strategies and their association with the *COMT* Val/Met genotype. A significant difference between the coping scores of homozygous G/G and A/A individuals was found (Wilcoxon Rank-Sum test: z = −2.20, *η*^*2*^ = 0.6, p = 0.02, n = 9). Individual data points are represented using solid dots. Boxes represent interquartile ranges, and whiskers represent the upper and lower limits of the data. The horizontal bars within the boxes represent the median values.

We analyzed a subset of individuals for whom *COMT* polymorphism information was available, and a specific coping style could be assigned (n = 18). Due to a small sample size, we could only compare homozygous A/A (n = 6) and G/G (n = 3) individuals from the aggression-based coping category and found a difference (Wilcoxon Rank-Sum test: z = −2.20, *η*^*2*^ = 0.6, p = 0.02, Figure 4). Homozygous G/G individuals (1.07 ± 0.19) had significantly higher coping scores than their homozygous A/A counterparts (0.60 ± 0.15). This was in contrast to the personality traits, which did not correlate with the genetic polymorphism (see Figure S4). Finally, if we visually inspect the thermography data, it is interesting to see that the two individuals with the G/G genotype of which we also had thermal data were the ones that recovered most (marked by high nose temperatures) from the stressor (see Figure 2).

## Discussion

We investigated coping mechanisms in an NHP species using a multidisciplinary research design. We found two non-correlating aggression- and nonaggression-based coping factors, characterized by direct aggressive engagement with stressors and conspecifics, and exhibition of affiliation toward conspecifics, respectively, during predator exposure experiments. Further inspection revealed aggression-based, nonaggression-based, mixed coping styles, and low coping reactivity in these animals. Aggression-based copers exhibited lower emotional arousal than nonaggression-based ones, as examined using a non-invasive infrared thermal imaging method. Personality traits partially predicted coping, particularly the nonaggression-based coping style, where higher affiliation was associated with lower nonaggression-based coping scores. We furthermore provided the first evidence of the human equivalent *COMT* Val/Met polymorphism in long-tailed macaques. Although identified in a few individuals only, we could show that the G variant in a homozygous state (G/G, two alleles encoding for Val) was associated with aggression-based coping. This suggests a genetic basis for a complex behavior, such as coping. Our findings underscore the importance of multidisciplinary approaches in understanding coping mechanisms and their evolution.

Our results align with previous studies on coping styles and associated variations among individuals.^15^^,^^19^^,^^32^^,^^33^^,^^37^^,^^63^ However, unlike a proactive-reactive continuum,^15^ which is predominantly described in the non-human animal literature, we found non-correlating aggression- and nonaggression-based coping styles, suggesting independent coping strategies in long-tailed macaques (see^64^). The aggression-based coping style was associated with direct engagement with the stressor through aggressive behavior and also the exhibition of aggression toward conspecifics. Out of all the individuals to whom a specific coping style could be assigned (n = 22), we found ∼68% of them to have aggression-based coping styles. This is in line with the idea that a more despotic species, such as long-tailed macaques, would exhibit lower inhibition control than more socially tolerant species.^65^ On the other hand, the intensity of aggression toward conspecifics can be attributed to “frustration” and “redirection,” critical signs of societies with low social tolerance.^57^^,^^66^ The aggression-based copers retained higher average facial temperatures after an initial 10-min of predator exposure than their nonaggression-based counterparts, thus, possibly lower emotional arousal; it might indicate their predisposed neural underpinnings, which made them “proactive,”^67^ i.e., better equipped to engage and cope with stressful situations. In other words, the aggression-based copers were more efficient in controlling (albeit not voluntarily) emotional arousal than nonaggression-based copers with regard to the activities of the autonomic sympathetic nervous system. On the other hand, the nonaggression-based coping dimension had positively loaded variables of close (distance to stressor) and conspecific affiliation and negatively loaded variable of far (distance to stressor). Even though the variables close and far were not mutually exclusive (see Table S1), they seem to have covaried. Yet interestingly, it suggests that individuals with nonaggression-based coping styles were not entirely avoiding the stressor but, at the same time, not directly engaging with it like their aggression-based counterparts. These results suggest the complex and multifaceted structure of coping and the less distinctive extremes of its strategies.^14^^,^^34^ However, these individuals also seemed to rely on support seeking through affiliation from conspecifics, which might act as “social buffering.”^68^^,^^69^^,^^70^ Thus, a nonaggression-based coping strategy might also resemble the emotional coping style and seem beneficial in long-tailed macaques, where close social bonds are prerequisites for maintaining group cohesion, cooperative interactions and sociality.^71^^,^^72^

A relatively new and non-invasive measure of infrared thermography provided information for emotional arousal in coping in our study. As opposed to the aggression-based, persistently lower facial temperatures in nonaggression-based copers were found, potentially indicating higher emotional arousal. Thus, the behavioral coping styles correlated with HPA axis reactivity. These findings corroborate that nonaggression-based copers require prolonged social support from conspecifics to reduce arousal and cope with stressors. While the apparent non-independence of behavioral coping styles with HPA axis reactivity may seem contrasting to the prediction of the two-tier model, it should be noted that thermography data from the low reactants could not be obtained. Although they neither significantly engaged in predator aggression nor relied on conspecific support, the missing information on emotional arousal has made it challenging to conclude whether behavioral coping styles and reactivity are (in)dependent components of coping. Nevertheless, they seem to have covaried. For example, while personalities did not affect coping styles in wild baboons, neuroticism was predicted by stress reactivity.^73^ Finally, despite controlling for obvious confounding factors (e.g., indoor/outdoor temperature differences, distance and angle of the subjects from the thermal camera, and so forth), other unprecedented factors (see^74^) can cause a reduction in the precision of the thermal measures. Therefore, a methodological comparison between infrared thermography and endocrine measures would be recommended to better understand the HPA axis reactivity in coping.

Using the comprehensive multi-method approach of behavioral observations and experiments, we found three personality traits in long-tailed macaques – activity-sociability, affiliation, and exploration. Traits similar to activity-sociability and affiliation had previously been identified in long-tailed macaques.^75^ While activity-sociability and affiliation in our study represented variables from an observational perspective, repetitive “rare” behaviors were captured using experiments in the exploration dimension. This validates the concept of objective assessment of personalities in non-human animals.^53^^,^^76^ Age and sex were found to influence personality traits. Age had a negative effect on activity-sociability and exploration. This effect may be attributed to the active and playful underpinnings of some loaded variables within those traits, e.g., *social play, handling container,* and *object manipulation*. Play behavior is essential for the locomotive, cognitive, and social development of young individuals.^77^ Therefore, younger individuals are expected to score higher on these traits than older individuals, in which activity budgets and priorities may have shifted more toward goals such as reproduction and competition.^78^ Alongside age, sex was also found to significantly predict personality scores on activity-sociability and exploration, in which males scored higher than females. This may be attributed to female philopatry and male dispersal in long-tailed macaques, where males tend to leave their natal group around the age of four years and join either a bachelor or a new social group.^79^ Although personalities, following a strict definition, should not be sex-dependent, there is growing evidence for sex-specific personality dimensions. Differential selection pressures and varying life history trajectories are considered the underlying mechanisms for the evolution of sex-dependent personality traits.^80^

We found partial support for our hypothesis of personalities predicting coping styles. The observed personality traits did not affect aggression-based coping styles. This indicates that the aggression-based coping style in itself may be an independent personality trait, equivalent to boldness, and the associated variables of our aggression-based coping may have captured boldness-related behaviors. However, it is also important to emphasize that we did not find any evidence of “boldness-explorative” behavioral syndromes, as reported in previous studies.^81^^,^^82^ Nonaggression-based coping, on the other hand, was inversely associated with personality trait affiliation. This again supports the social buffering hypothesis that (seeking) affiliative interactions can potentially alleviate the impact of stress. Macaque societies are characterized by strong positive relationships among individuals, such as friendships,^71^ thus presenting opportunities to seek support from group members in distress. This strengthens the effectiveness of close social relationships in reducing stress in group-living species. Interestingly, we noted that females scored higher than males in the nonaggression-based coping dimension. While this observed sex difference may be explained by a general tendency of females to be more anxious than males,^83^ and/or possibly female philopatry (see^79^), our results here should be interpreted with caution due to a low sample size of males. Contrary to our prediction, although linear and steep hierarchies in all three groups were noticed, we did not find any relationship between dominance-rank relationships and coping styles. A recent study on wild baboons (*Papio anubis*) also found no relationship between social hierarchies and coping styles.^84^ These results are unsurprising because dominance and coping styles are group- and individual-level properties, respectively, and might not always covary.

In our final analysis, we were interested in whether potentially different *COMT* genotypes modulate differences in the coping styles of long-tailed macaques. In humans, a functional polymorphism located in exon 4 of the *COMT* gene (Val^158^Met) has garnered significant scientific attention due to its counterbalancing effect on emotional resilience, stress, anxiety and cognition.^60^ Even though the catecholaminergic system appears highly conserved among vertebrates, the *COMT* gene has received little attention in NHP studies. To our knowledge, the Val/Met polymorphism has only been reported in Assamese macaques^38^ among *Macaca* and thus has not been studied extensively regarding emotional arousal or coping styles in NHP species. We found an association between the *COMT* Val/Met polymorphism and aggression-based coping style; G/G individuals (Val/Val) scored higher on coping than individuals carrying the A/A (Met/Met) variant, as hypothesized.^62^ This finding suggests a potential genetic basis for coping styles in long-tailed macaques.^85^^,^^86^ Our results align with the “warrior-worrier” model used to explain the existence of two alleles that induce distinct behavioral phenotypes in humans. The model claims that the Val-allele confers an advantage in confronting aversive stimuli (greater stress resilience and lower anxiety levels, i.e., warrior), whereas the Met-allele is beneficial in cognitive tasks (e.g., memory and attention tasks; worrier). Due to a low sample size, driven by just one individual carrying the G/G variant, comparisons could not be made among nonaggression-based copers, which would be an important avenue for future studies. In addition, as highlighted, the two least emotionally aroused individuals (as measured by infrared thermography) in our study were aggression-based copers with the G/G variant, potentially indicating an effect of *COMT* Val/Met polymorphism on emotional arousal. To what extent the *COMT* Val/Met polymorphism can influence animal emotion would thus be interesting to explore in future. It is, however, crucial to highlight that the same polymorphism had no relationship with the observed personality traits in this study, potentially indicating non-overlapping genetic underpinnings of coping styles (and potentially emotionality) and personalities.

The candidate genes of the serotonergic and dopaminergic systems are known to mediate coping in humans^87^ and thus may explain the underlying causal mechanisms. However, as pointed out by earlier studies, there is an urgent need to consider the candidate genes while understanding the causal mechanisms of coping. Similar results in humans and long-tailed macaques regarding the *COMT* Val/Met polymorphism and coping further highlight an evolutionary basis of this mechanism and stress regulation in general. Although the effect of Val/Met on human behavior has been intensively studied, we know that findings on the contribution of single candidate genes to a complex behavioral trait must be interpreted with caution.^88^^,^^89^ We call out for the replication of our study, which is the first to reveal an impact of *COMT* on coping behavior in an NHP species. Genome-wide association studies can help decipher the involvement of other gene variants, thereby providing a more detailed picture of the interaction of underlying genotype and coping behavior. In addition, comparative studies on other species may reveal the evolutionary history of these traits and their genetic bases.

Using a comprehensive research design, we provided evidence of two non-correlating coping factors in a group-living NHP species. We identified the complex interplay of proximate mechanisms pertaining to behavior, physiology (emotional arousal) and genetics. The complexity and dimensions of coping can be wide and should be investigated carefully. While varying concepts and methodologies are currently being applied, multidisciplinary research designs with novel methodologies are crucial to understanding the overarching process of coping. Although species-typical responses can be at play, identical research designs should be applied to a wide range of taxa to get a grip on the evolution of coping.

### Limitations of the study

Although we employed a relatively novel infrared thermography approach to measure emotional arousal, the methodology has some limitations. Relatively large time windows were chosen to investigate temperature differences. This step enabled us to increase the number of individuals for analyses, but at the cost of precise temperature detection in much shorter windows. Also, for inclusion in the thermography analyses, we only relied on individuals who approached close to the fences, indicating a potential sampling bias. However, this is true for most behavioral studies conducted in group settings, where dominant, bold and explorative individuals are potentially chosen as sampling subjects. Nevertheless, employing two thermal cameras during the experiments to maximize sampling effort, and thermal data on individuals with different coping styles (albeit posthoc) suggests that our experiments and the results were not greatly affected by this bias. Furthermore, as mentioned above, a methodological comparison should be carried out between infrared thermography and endocrine measures in quantifying the emotionality of coping. The sample size of the aggression- and nonaggression-based copers with the different *COMT* Val/Met genotypes was very small, but it depended on the allele frequencies. Besides, while we first report the presence of a human equivalent *COMT* Val/Met polymorphism in long-tailed macaques, complex behavioral mechanisms, such as coping, may have complex gene interactions. Therefore, the replication of our study with a larger sample of animals should be conducted, and other potential candidate genes must be included in the research design to check for any interactive effects of those genes.

## STAR★Methods

### Key resources table

**Table:** 

REAGENT or RESOURCE	SOURCE	IDENTIFIER
**Biological samples**		

gDNA from EDTA whole blood samples *Macaca fascicularis*	This study	N/A

**Chemicals, peptides, and recombinant proteins**		

Blood tube 3ml.vac.EDTA	Greiner Bio-One BV	Cat#454086
ELB (Erythrocyte lysis buffer):		
NH_4_Cl	Merck Life Science N.V.	Cat#168320
KHCO_3_	Merck Life Science N.V.	Cat#104854
EDTA	VWR International BV	Cat#1.08452.1000
TE^4^-buffer:		
Tris Base	VWR International BV	Cat#648311-5kg
EDTA	VWR International BV	Cat#1.08452.1000
Proteinase K	Fisher Scientific B.V.	Cat#EO0492
SDS	Sigma Aldrich	Cat#13771-1kg
NaCl	VWR International BV	Cat#27810.262
2-propanol (Isopropanol)	VWR International BV	Cat#MERC1.09634.2500
70% EtOH (made from 100% EtOH)	VWR International BV	Cat#1.00983.1000
Buffer B2	Solis BioDyne	Cat#01-02-00500
MgCl_2_ (2.5 mM)	Solis BioDyne	Cat#01-02-00500
dNTP’s (0.2 mM)	ThermoFisher Scientific	Cat#R0242
EvaGreen®	Biotium	at.VWR.com; Cat#31000-T
FIREPol hot-start DNA polymerase	Solis BioDyne	Cat#01-02-00500

**Critical commercial assays**		

Omega E.Z.N.A. Gel extraction kit	Omega Bio-tek	at.VWR.com; Cat# D2500-01

**Deposited data**		

Raw data and code	This study	https://osf.io/v4uef/?view_only=e8781c3e8052404bb7635aaf4c99afee
SNP data generated in this study	ENA - EMBL	Project Number: PRJEB65031

**Experimental models: Organisms/strains**		

Long-tailed macaques (*Macaca fascicularis*)	Biomedical Primate Research Centre, Rijswijk, the Netherlands	N/A

**Oligonucleotides**		

Primer COMT: forw: 5′AAGATCGT GGACGCCGTG	Pflüger et al., 2016	https://doi.org/10.1016/j.yhbeh.2015.11.012
Primer COMT: rev: 5′ACAGTGGG TTTTCAATGAACGTG	Pflüger et al., 2016	https://doi.org/10.1016/j.yhbeh.2015.11.012

**Software and algorithms**		

CodonCode Aligner software, version 10.0.2	CodonCode Corporation	codoncode.com
R (version 4.3.1)	R Core Team	https://www.Rproject.org/
Adobe Illustrator (version 25.3)	ADOBE	https://www.adobe.com/products/illustrator.html
FLIR Tools (version 6.4.18039.1003)	Teledyne FLIR LLC	https://www.flir.eu/products/flir-research-studio/?vertical=rd%20science&segment=solutions

### Resource availability

#### Lead contact

Further information and requests for resources should be directed to and will be fulfilled by the lead contact, Debottam Bhattacharjee (bhattacharjee.debottam@gmail.com).

#### Materials availability

This study did not generate new materials.

#### Data and code availability

• Data: All data generated in the study have been deposited at Open Science Framework and are publicly accessible at https://osf.io/v4uef/?view_only=e8781c3e8052404bb7635aaf4c99afee, which is also listed in the key resources table. SNP data have been submitted under ENA-EMBL project number PRJEB65031, which is also listed in the key resources table.
• Code: All original code used in the study have been deposited at Open Science Framework and are publicly accessible at https://osf.io/v4uef/?view_only=e8781c3e8052404bb7635aaf4c99afee, which is also listed in the key resources table.
• Any additional information required to reanalyze the data reported in this paper is available from the lead contact upon request.

### Experimental model and study participant details

We studied three groups of captive long-tailed macaques (*Macaca fascicularis*) housed at the Biomedical Primate Research Centre (BPRC) in Rijswijk, the Netherlands. The first group (Gr.1) consisted of 15 individuals; four were under the age of one year at the beginning of the study and were not included (n = 11). The remaining 11 individuals consisted of seven females and one male, all above the age of three years, and two females and one male between the ages of one and three years. The second group (Gr.2) originally consisted of 18 individuals but was reduced to 17 as a male was removed early during the study due to compatibility issues with other group members (n = 17). The remaining 17 individuals included ten females and one adult male above three years, and two females and four males aged between one and three years. The third group (Gr.3) comprised four males, all aged above three years (n = 4). A detailed description of the individuals is provided in Table S8.

Gr.1 and Gr.2 had continuous access to indoor and outdoor enclosures with two or more interconnected compartments each. Gr.3, on the other hand, had a single indoor and outdoor compartment access. For Gr.1 and Gr.2, the indoor and outdoor enclosures were approximately 49 m^2^ and 183 m^2^, respectively. Gr.3 had an indoor and outdoor enclosure of about 3,55 m^2^ and 3,88 m^2^, respectively. Immediately adjacent to the enclosures were other groups of long-tailed macaques that were partly visible. However, the three study groups had no visual contact with each other. They were kept at distant buildings or in the same building but on two different floors (like Gr.1 and Gr.2). All enclosures had multiple enrichment structures, including slides made from firehoses, plastic toys, wooden structures of various heights, platforms, climbing stairs, a plastic pool (except for Gr.3), and a tree trunk. Note that the exact enrichment materials may have differed to some extent across enclosures. Extra enrichment materials, such as branches and paper containers, were provided when available. The indoor enclosure had concrete floors covered with sawdust bedding, while the outdoor enclosures were covered with natural soil and sand substrates. All indoor enclosures were temperature controlled and maintained a constant temperature of 22°C. The feeding of the animals always took place in the indoor enclosures. The diet consisted of monkey pellets in the morning, placed in feeding cans attached to the enclosure fences, and vegetables in the afternoon, with an occasional seed mix (corn, sunflower, etc.) being thrown in to stimulate foraging behavior. Water was available 24/7 *ad libitum* in both indoor and outdoor enclosures.

The BPRC is accredited by the Association for Assessment and Accreditation of Laboratory Animal Care (AAALAC) and licensed to keep non-human primates for ethological and biomedical research. The institution follows high standards of animal welfare and refinement measures. Our study complied with all ethical regulations and guidelines for animal testing of BPRC’s Animal Experiments Committee and Animal Welfare Organisation (Animal Welfare Organisation/IvD approval no. 019E). In addition, during the studies, at least one experimenter was present who was trained and certified (FELASA Accredited Laboratory Animal Science 066/19AF) to conduct animal experiments in accordance with the requirements of Article 9 of the Dutch Experiments on Animals Act.

### Method details

#### Coping

We conducted predator model experiments repeatedly to investigate coping. Two life-sized predator models were used, and the experiments were conducted in two phases, with an interval of at least two months in between. The order in which the experiments were carried out across groups was randomised. The first model was a snake (160 cm long and resembling a python; Figure S5A), and the second was a bird of prey (resembling a hawk with a wingspan of 70 cm; Figure S5B); both of which are natural predators of long-tailed macaques in the wild.^90^^,^^91^ The snake model had a coiled posture, known to elicit threat responses more than a sinusoidal posture,^92^ whereas the bird had a standing, wings-up position. The models were placed out of reach of the individuals (∼60 cm away from the enclosures), in front of the indoor enclosures for Gr.1 and Gr.2, and in front of the outdoor enclosure for Gr.3. We made sure that the neighbouring groups had no visual access to the predator models, either by blocking the corridors with natural tree branches (Gr.1 and Gr.2) or placing the models inside a 180 cm x 70 cm x 70 cm wooden box with only the front exposed to the to be tested group (Gr.3). All groups were habituated to these modifications (without the predators) on the days before testing for at least 24 hours. Besides, on the days of testing, the immediate next enclosure compartments belonging to the other groups were emptied. This is standard practice during enclosure cleaning and maintenance at the BPRC; therefore, we assume that individuals from the focal groups will react similarly on testing and non-testing days. The predator exposure experiments lasted half an hour and were recorded using two video cameras (Canon Legria HF G25 and Sony FDR AX100E 4K) mounted on tripods from different angles.

The facial (nose bridge, tip and mid-nose) temperature data of the subjects were collected using infrared thermography (cf.^93^) throughout the predator exposure experiments. Facial temperatures were collected using two FLIR E96 (640 x 480 thermal resolution) thermal cameras by two experimenters who either sat on the ground (for Gr.1 and Gr.2) or stood (Gr.3) at least 1 metre away from the enclosures. The emissivity was set at 0.97; typically, a value of 0.97 or 0.98 is used for non-human primates.^94^ Reflective- and ambient temperatures and humidity measures were set at the thermal cameras before using them. We used two (for reliability) ThermoPro TP50 thermo-hygrometer devices to record ambient temperature and humidity. Any ambient temperature and humidity changes were noted and subsequently adjusted in the thermal cameras during the experiments. All subjects were habituated to the presence of the experimenters for over a month and at least a week to the thermal cameras. Three key criteria were followed to collect reliable facial temperature data to assess emotional arousal: (i) the distance between the experimenter and an individual subject is within 2 meters, (ii) an individual is facing the thermal camera at an angle not more than 50°, (iii) an individual is not moving abruptly and staying relatively still (cf.^27^^,^^56^^,^^93^^,^^94^).

#### Personality

Personality was assessed using a multi-method bottom-up approach consisting of behavioral observations and novelty experiments. A multi-method process is expected to deliver a more complete view of personality objectively than each method separately.^95^ Following an extensive and standardised ethogram,^53^ we used a continuous focal sampling method for the behavioral observation part. To investigate the temporal consistency in behavior, being a prerequisite for personality traits,^28^ focal observations were conducted in two different phases: for Gr.1, the first phase took place from March to July 2022 and the second from August to September 2022; in Gr.2, the first phase spanned between February and June 2022, and the second from August to November 2022. In Gr.3, focal observations were conducted over one relatively long temporal window from November 2021 to April 2022. These observations were later divided into two non-overlapping phases to check for temporal consistency. Each focal follow was 20 minutes long and recorded with a video camera (Canon Legria HF R806), handheld or mounted on a tripod. The order of sampling was pseudorandomised, and no individual was observed consecutively. Focal sampling took place 3-4 days a week, between morning (0900 and 1200 hours) and afternoon (1201 and 1500 hours) feeding schedules of the monkeys. Each focal animal was observed during a morning and an afternoon session on the same day, and both sessions were equally sampled.

The experimental approach entailed exposing the individuals in their social group setting to three categories of novelties - food puzzles, novel food items, and novel objects (see Figure S6). Each category had two types (i.e., two types of food puzzles, two types of novel objects, etc.) to check for contextual consistency in behavior. Similar to the focal observations, the personality experiments were conducted in two different phases, rendering the commencement of a total of 12 experiments per group. For Gr.1, the first phase of experiments occurred from January to February 2022 and the second from May to July 2022. For Gr.2, phase one was carried out between April and July of 2022 and phase two between August and September 2022. For Gr.3, the first phase occurred between December 2021 and February 2022 and the second from August to November 2022. Only one experiment was conducted on a given day; their order was pseudorandomised between the two phases. Experiments belonging to the same category were not conducted consecutively. In addition, observations and experiments were conducted on different days for a group. We performed all the experiments in the indoor enclosures except for Gr.3, where experiments took place in both indoor and outdoor enclosures. For each experiment, the animals were locked briefly in their outdoor (applicable to all) or indoor (applicable to Gr.3) enclosures while the task was being set up. At the BPRC, all macaques are trained to move to outdoor or indoor enclosures by voice commands of the caretakers. Therefore, movement before the experiments most likely caused no additional stress to the animals, and which is assumed to be comparable to non-testing days. All experiments were recorded using video cameras (Canon Legria HF R806, Canon Legria HF G25, and Sony FDR AX100E 4k) mounted on tripods. Each camera focused on a specific compartment of Gr.1 and Gr.2 enclosures. For Gr.3, due to its small enclosure area, two cameras were placed, one focusing on the indoor and one on the outdoor enclosure. Video recording was started before the animals regained full access to their enclosures and ended when the experiments were over.

We used two types of food puzzles: a box and a pipe. A wooden maze box (∼30 cm x 40 cm x 15 cm; see Figure S6A) with a plexiglass front cover was used, which had two narrow horizontal openings, enabling individuals to move the food rewards (cut pieces of apples) with their fingers. Solving the puzzle required animals to move the reward far enough to the left so that it would roll downwards to the right. At that point, it would have to be moved to the left again towards the opening from where it could be retrieved. During each experiment, three identical boxes containing multiple cut pieces of apples were secured to the bars of the indoor enclosure, roughly 15-20 cm above the ground. The distance from the ground allowed enough space for the apples to fall out. For Gr.3, considering the smaller group size, we used two boxes instead of three. Multiple box puzzles (similarly applicable to other objects/items) helped reduce monopolisation.

The pipe puzzle (∼70 cm in length, Ø 15 cm; see Figure S6B) was made of hard plastic and had a row of small holes (∼Ø 2 cm) on the upper side. During each experiment, three and two identical pipes, for Gr.1/Gr.2 and Gr.3, respectively, containing three handfuls of a seed mix (corn, sunflower, etc.), were hung up roughly 100 cm from the ground. The food rewards could be retrieved by rotating and holding the pipe far enough for the rewards to fall downwards through the holes. The tension of the strings assisted the pipes to return to their original positions when released. Apart from multiple identical items, the puzzles were placed in a way (e.g., in different compartments) that potentially minimised monopolisation by higher-ranking individuals. The experiments started with monkeys regaining access to the enclosures and ended either after one hour or after all puzzles had been solved (i.e., all rewards were obtained), whichever was earlier.

The novel food items used during the first phase of Gr.1 and Gr.3 were rambutans (see Figure S6C) and dragonfruits (see Figure S6D). However, due to veterinary advice and reconsideration, the rambutans were replaced with starfruits during the second phase of experiments. The monkeys had no experience with either of these fruits. In Gr.2, starfruit and dragonfruits were used in both phases. During each experiment, multiple pieces of either intact (rambutans) or halved (dragonfruit and starfruit) fruits were placed in the enclosure ground. The number of novel food items was adjusted according to the group sizes to avoid monopolisation. Like food puzzles, the experiments started with monkeys regaining access to their enclosures and ended after one hour or when all items were eaten.

The two novel objects used were blue and green coloured plastic egg containers (14 cm x 13 cm x 4 cm; see Figure S6E) and wooden massage rollers (24 cm x 10 cm x 4 cm; see Figure S6F). For Gr.1 and Gr.2, six identical objects were used during each experiment and spread about 100 cm apart from each other in the indoor enclosure compartments. In comparison, we used two objects of each type for Gr.3. Since the individuals could carry the objects freely, we recorded the monkeys‘ activities in indoor and outdoor enclosures. Each experiment lasted for an hour.

#### Collection and storage of blood samples for genetic analyses

EDTA whole blood samples were obtained from the monkeys during annual veterinary health check-ups to isolate genomic DNA (gDNA) using a standard salting-out purification procedure. The check-ups are distributed over the year and have the target of sampling each animal with an interval of approximately 12 months. The individuals in our study did not show any clinical signs of diseases based on daily care and observations. Besides, monkeys were only included in the annual veterinary health check-ups when they were ≥ 9 months old. The collection of blood samples took place in the morning, and monkeys did not receive food after 1700 hours on the previous day. Progressive refinement procedures were followed to minimise the stress of capture before the sedation process. Monkeys were trained to voluntarily enter the squeeze cage as a part of the refinement procedure. After an intramuscular injection of the sedative, blood sampling was carried out by qualified animal caretakers within a window of 20 minutes. The collection site was sterilised using 70% alcohol. The monkeys gained consciousness within 2 hours of the procedure. Notably, no behavioral experiments or observations were conducted within a week of the health check-ups.

From each monkey in the studied cohort, 30 μl of gDNA with a concentration varying between 46-649 ηg/μl (on average 124,5 ηg/μl) was shipped to Affenberg Landskron Field Research Lab (field research station of the University of Vienna; Landskron, Carinthia, Austria) for further analysis.

### Quantification and statistical analysis

#### Behavioral coping styles

Self-directed behaviors are considered proxies for quantifying stress-related responses in non-human primates (see,^96^^,^^97^ but see^98^^,^^99^). To investigate whether the predator models indeed played the role of stressors, we first compared self-directed behaviors (autogroom and scratch combined) between predator exposure experiments (all phases and predator models combined) and regular focal data (phases combined). Frequencies of self-directed behaviors were calculated per minute at the individual level. We calculated the frequencies from focal data when no aggressive interactions were observed for at least five minutes to eliminate any potential effect of social stress.

Videos were coded using Solomon Coder (version beta 17.03.22). The durational (s/hour) and event behaviors (frequencies/hour) were calculated per individual and were corrected for the time spent out of sight. We set up a minimum threshold of 6 minutes of within-sight observation (10% of the total combined observation time/phase) for the individuals to be considered for analyses. Despite attempts to record all individuals with multiple cameras, we could not obtain adequate data from two individuals (two females above three years of age) belonging to Gr.2. Therefore, we revised our sample size for the behavioral part of coping from 32 to 30 individuals. In addition, even if two predators could elicit responses from monkeys quite differently (see^54^), we decided to combine their data for each phase (at the individual level) to tackle the low occurrences of some of the variables. Three experimenters coded the behavioral coping videos, and inter-rater reliability was calculated. It was found to be high (based on 33%, i.e., 4 out of 12 predator exposure videos; (ICC (3,k) = 0.97, p < 0.001).

The consistency of the behavioral coping-related variables was examined using a two-way mixed model intraclass correlation (ICC (3,1)) analysis. Only variables with sufficient temporal consistency (ICC values ≥ 0.3 and p < 0.05) were included in further analyses. ICC analysis yielded 9 repeatable variables with ICC values ranging from 0.32 to 0.80 (see Table S9). These repeated variables (average values between phases one and two) were used in the EFA with a principal axis factoring (PAF) extraction method. PAF was chosen as it does not assume a multivariate normal distribution, and the data might exhibit significant deviation from a normal distribution. After an initial run of the EFA, four variables, *foraging, locomotion, freeze,* and *yawn,* were removed due to low Kaiser-Meyer-Olkin measure of sampling adequacy (MSA) scores. The final model included 5 variables with an overall acceptable MSA score of 0.62. Bartlett’s score of sphericity was significant (x^2^ = 41.56, p < 0.001); thus, the assumptions of EFA were met. The latent factors were then extracted based on eigenvalues >1. The root mean square of the residuals showed a value of 0.02, which indicated a good model fit. In addition, a Tucker-Lewis Index (TLI) value of 0.95 was obtained, further suggesting that the model fit was sufficient.^100^ We performed a promax rotation method for meaningful interpretation of the potentially correlated factors and factor loadings. The resulting factors were labelled based on which behavioral variables loaded significantly (≥ 0.5, positive and negative) onto them (i.e., coping styles^12^^,^^15^^,^^34^^,^^37^). The scores of each individual per factor were extracted. Due to a relatively small sample size, instead of k-means clustering (see^101^), we decided to perform the categorization of individuals into different coping styles using a visual inspection followed by a median split approach (cf.^64^). The median split approach, in particular, allowed us to employ a conservative categorization. Factor scores were assigned to individuals only if they scored higher than the factor median. Moreover, between-factor comparisons were made using Wilcoxon signed-rank tests before assigning them to the individuals as specific coping scores. Effects of sex and age were checked on the obtained coping styles.

#### Emotional arousal

Following the three criteria of collecting thermal images, we extracted information from the individuals across all phases and predator models. The number of thermal images and individuals was adjusted based on the specific coping styles of the monkeys. One experimenter coded all the thermal recordings; another experimenter coded 5% of the images (∼64 images, including whenever individuals were visible) obtained from the videos to check for reliability; it was found to be high ((ICC (3,k) = 0.93, p < 0.001). Not all individuals were equally sampled, as the procedure heavily depended on monkeys approaching the enclosure fences. The valid thermal images were imported to FLIR Tools (version 6.4.18039.1003) for analysis. Nose-bridge, tip, and mid-nose temperatures were extracted from each image. Each image was magnified (606%) consistently, and an area size of 2 x 2 pixels was applied to the designated regions to extract temperature measures (see below figure). Temperature measures from the three regions were then checked for correlation using Spearman Rank correlation tests. High correlations between the nose bridge, tip, and mid-nose regions for facial temperature readings (Spearman's Rank Correlation: nose tip and mid-nose, rho = 0.99, p < 0.001; nose-tip and nose bridge, rho = 0.99, p < 0.001, mid-nose and nose bridge, rho = 0.99, p < 0.001) was found. Since the mid-nose region is less susceptible to temperature changes due to breathing, mid-nose temperature data were used for the analysis (see^93^).

**Figure:**
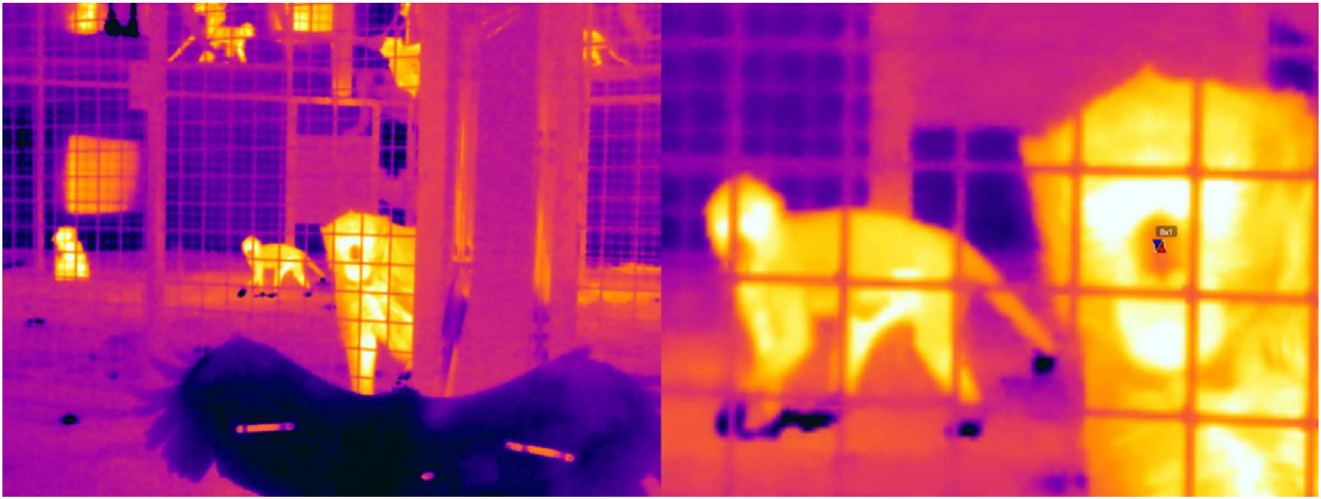
Infrared thermal imaging to measure emotional arousal The left panel shows the experimental set-up using the predator hawk in one of the long-tailed macaque groups. The right panel shows a zoomed-in version (606%) where the mid-nose temperature is extracted from a focal individual using a 2 x 2-pixel area. The pixel area provides an upper (up arrow), a lower temperature (down arrow), and an average estimate. We used the average value for analysis.

Even though the animals were trained to move between in-/out-door enclosures following the commands of caretakers, we could not discard the possibility of emotional arousal associated with this process. Therefore, instead of using potentially unreliable thermal measures (as control) before the introduction of the predator, we decided to divide the 30-min predator exposure period into small time windows for comparison. We did not code data for the first two minutes of the predator exposure to avoid any potential influence of temperature differences between the enclosures and emotional arousal due to the associated lock and release. Additionally, if an individual travelled, during the experiment, between the indoor and outdoor enclosures, a 1-min no coding window for the individual was applied to control for potential temperature differences; besides, data were not coded for 1-min if a focal individual was found vocalising (cf.^27^^,^^56^^,^^93^). We divided the overall duration of the experiments into the following time windows: 2-10 min, 10-20 min, and 20-30 min. Such a division ensured maximised sampling effort from the individuals. Individuals were only included in the analysis if at least one valid data point was collected from each time window. In case multiple data points were found pertaining to one individual within a time window, the average value was used. Since the aim was not to determine how quickly the facial temperature changed upon predator exposure but rather how coping strategies worked throughout the session, we converted all temperature values of the individuals to '0' from the 2-10 min window. This was used as the baseline measure. However, this is not to say that we did not expect a potential drop in temperature upon predator exposure. Temperature changes in the following periods (i.e., 10-20 min and 20-30 min) were compared with the baseline. Finally, we conducted a validation test using locomotion and foraging behaviors to control for the potential confound of the general activity of the monkeys. Following the inclusion criteria, we could extract reliable data on emotional arousal from 12 individuals (six each for aggression- and nonaggression-based coping styles).

#### Personality

We collected data on all 32 individuals. A total of 10,229 minutes of focal data (Phase 1: mean ± standard deviation = 159.76 ± 2.67 observation minutes/individual; Phase 2 = 159.89 ± 2.64 min/individual) was collected in addition to the 36 personality experiments from the two phases. Four coders coded observational and experimental videos, and inter-rater reliability was estimated using a two-way mixed ICC coefficient (ICC 3,k = 0.96, p<0.001). We used 5% of the observational and experimental data to calculate the inter-rater reliability.

For each phase of the focal observation, durational variables (sec/min) and event behaviors (frequencies/min) were calculated per individual, and the varying observation minutes were corrected. The variables for each category of the personality experiments were coded (also see ^53^) as follows: *latency to approach* (sec) as the time it took for an individual to move from an initial 5-meter distance to a 1-meter radius of the novel object, novel food, or food puzzle. If no approach was made, *latency* was scored as the total length of the experiment; *proximity* (s/hour) as the total time an individual spent in proximity (≤ 1 meter) of the novel object, novel food, or food puzzle; *manipulating* (s/hour), or *handling* (s/hour), as the time an individual spent manipulating or handling the food puzzle or novel object, respectively. The coding of *proximity* was seized as soon as an individual began *manipulating* or *handling* the experimental items. All variables were standardised for meaningful interpretation of the next steps of analyses.

All three groups were used together to view the species-level personality constructs comprehensively. The temporal consistency of the variables was tested using a two-way mixed model ICC, which compared each observational and experimental variable between phases one and two. A conservative cutoff was set in which only variables with ICC values ≥ 0.5 and p < 0.05 were considered temporally repeatable. Variables in which over half (i.e., more than 16 individuals) of the individuals had zero occurrences were excluded.

We found 33 repeatable variables from the ICC analysis with values ranging from 0.50 to 0.95. The average values of these variables between phases one and two were included in a PCA, but the number was further decreased to 17 after removing those with low communality scores (cut-off value = 0.7) and to even 12 after removing those loading in more than one component with similar magnitude. The communality scores of the remaining 12 variables ranged between 72.28% and 94.14%, with an overall Kaiser-Meyer-Olkin measure of sampling adequacy (MSA) value of 0.81 (see Table 1). A scree plot was generated using an unrotated PCA, and the eigenvalue of each potential principal component was reviewed alongside the percentage of variance explained individually and cumulatively. The number of principal components was subsequently decided on by inspecting the plot, eigenvalue scores (>1), and the amount of variance explained cumulatively (>70%). Since personality traits can theoretically be correlated and form behavioral syndromes, we used a direct oblimin rotation technique. Factor loadings ≥ 0.5 (for both positive and negative loading) for the variables were considered significant. Finally, variables were removed if loaded on multiple components with similar magnitude. The resulting principal components were labelled based on the significant behavioral variables loaded on them. The scores of each individual per component, or personality trait, were extracted. Effects of sex and age on the personality traits were investigated.

#### Dominance hierarchy

Dominance rank relationships were calculated at the group level using a Bayesian Elo-rating method.^102^ The method was based on calculating winning probabilities, and we particularly used submissive behaviors coded from focal observations (*avoid, be displaced, silent-bared teeth, flee,* and *social presence*, see^53^) to assess the steepness of hierarchies. We checked for the independence of the above behaviors from personality assessment. After constructing the hierarchies, individuals were plotted according to their respective estimated Elo values (see Figure S7).

#### COMT genotyping

We genotyped our study animals (*n=26*) for the equivalent site of the human Val^158^Met encoded polymorphism located in exon 4 of the *COMT* gene. However, in rhesus macaques, the *COMT* gene encodes a 270 amino acid (aa) long product, which is one aa shorter as compared to the human COMT protein. As such, the human *COMT* Val^158^Met encoded polymorphism in macaques is located at codon 157.^52^ Amplification of the target site and sample preparation for sequencing was conducted at the Affenberg Landskron Field Research Lab (field research station of the University of Vienna; Landskron, Carinthia, Austria). Amplification products were outsourced for sequencing to LGC genomics (Berlin, Germany).

We used primers of Pflüger et al. (^51^) to amplify a 288 base pair product covering the target site (forw: 5′-AAGATCGTGGACGCCGTG, rev: 5′-ACAGTGGGTTTTCAATGAACGTG). Amplification was performed in duplicates along with two non-template controls using a MIC qPCR cycler (Bio Molecular Systems, Coomera, Australia). The 20 μl qPCR reaction contained nuclease-free H_2_0 (ThermoFisher Scientific, Geel, Belgium), Buffer B2 (1x conc., Solis BioDyne, Tartu, Estonia), 2.5 mM MgCl_2_ (Solis BioDyne, Tartu, Estonia), 0.2 mM dNTP’s (Thermo Fisher Scientific Inc., Waltham, USA), 0.2 μM of each primer (Integrated DNA Technologies, Leuven, Belgium), EvaGreen (1x conc, Biotium, Inc. 46117 Landing Parkway Fremont, CA 94538), 0.05 U/μl hot-start DNA polymerase (HOT FIREPol® DNA polymerase, Solis BioDyne, Tartu, Estonia), and 2 μl template DNA per reaction tube. Reactions were prepared in a sterile UVP PCR2 Workstation (Analytik Jena GmbH, Jena, Germany).

We cycled 15 min at 95°C, following 40x 10 sec at 95°C, 40 sec at 60°C and 20 sec at 72°C. To ensure that only a single product resulted from amplification, we included a melting curve analysis ranging from 72°C to 95°C with 0.3°C/s. The experimental samples showing a single peak at the expected melting temperature were purified using the Omega E.Z.N.A. Gel extraction kit (Omega Bio-tek, Inc. Norcross, USA). The spin column was loaded with 20μl PCR sample mixed with 20μl binding buffer. Duplicates of the same experimental sample were pooled. For purification, we followed the manufacturer’s instructions with only slight modifications. We added another washing step and refrained from loading the samples on an agarose gel to secure DNA yield. We used the melting curve results to verify the amplification products, and 10 μl of each sample plus 4 μl of forward primer were sent to LGC genomics. The resulting Sanger sequencing electropherograms were evaluated using the CodonCode Aligner software (version 10.0.2; CodonCode Corporation, Dedham, USA). Three additional non-synonymous SNPs were identified downstream of the target site. These polymorphisms were not linked to the target and, therefore, not subjected to further analyses.

#### Statistical models and packages

All statistical analyses were conducted in R (version: 4.3.1).^103^ Generalised linear mixed-effect model (GLMM) and linear mixed-effect model (LME) analyses were conducted using the *lmerTest*^104^ and *glmmTMB*^105^ packages. Null versus full model comparisons were done for all the models using the ‘lrtest’ function of the package lmtest.^106^ Model diagnostics were checked using DHARMa package.^107^ The multicollinearity of the fixed effects was examined with the help of the performance package,^108^ and a variance inflation factor of < 4 was set as a threshold for low correlation. Model selection was made based on the Akaike Information Criterion (ΔAIC = 2 as threshold) value using the ‘anova’ function. The significance value was set as 0.05 for all statistical tests, except for post hoc pairwise comparisons, where adjusted p-values were used following a Bonferroni correction method. See supplemental information for details on statistical model outputs (see Tables S3–S7).
