# Supplementary material for: Behavioral, physiological, and genetic drivers of coping in a non-human primate
Source: iScience. 2024 Jan 12;27(2):108890. doi: 10.1016/j.isci.2024.108890 (PMC10838955; doi:10.1016/j.isci.2024.108890)

iScience, Volume *27*

Supplemental information

Behavioral, physiological, and genetic drivers of coping in a non-human primate

Debottam Bhattacharjee, Aníta Rut Guðjónsdóttir, Paula Escriche Chova, Esmee Middelburg, Jana Jäckels, Natasja G. de Groot, Bernard Wallner, Jorg J.M. Massen, and Lena S. Pflüger

Figure S1 - Frequency of self-directed behavior, related to STAR Methods. The box plot shows the frequency of self-directed behavior (per minute) during baseline observation and predator exposure (Wilcoxon Signed-Rank test: z = -3.42, r = 0.66, p < 0.001, n=30). Individual data points are represented using solid dots. Boxes represent interquartile ranges, and whiskers represent the upper and lower limits of the data. The horizontal bars within the boxes represent the median values.

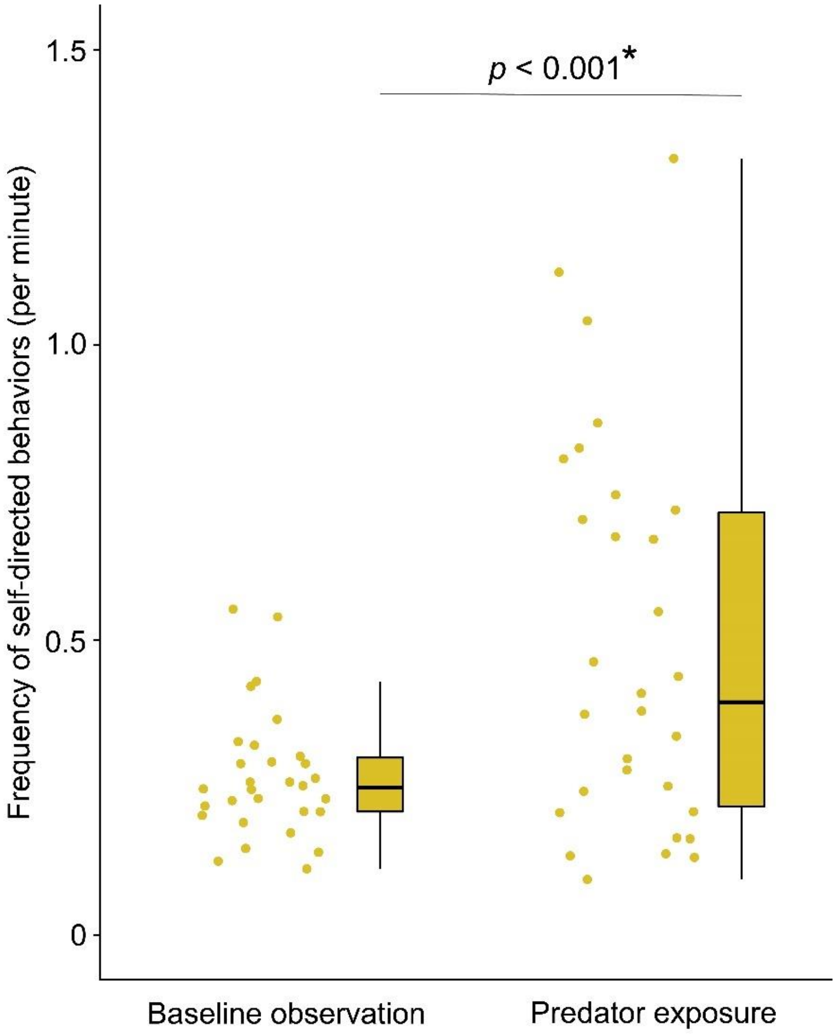

Table S1 – Ethogram of coping-related behaviors, related to STAR Methods. Name, type, and definition of all coping-related behaviors.

Behavior	Type	Definition
**Enclosure use**		
Close ground	State	Individual is within a 1-meter radius of the stressor and on the ground.
Far	State	Individual is outside of the 1-meter radius from the stressor and located either on the ground or on hanging structures of the enclosure. Or individuals within a 1-meter radius but on hanging structures but not on ground
**Activity**		
Locomotion	State	Individual walks or runs around, moves from one location to another, and is not stationary for more than 3 seconds.
Foraging	State	Individual moves slowly while looking for food on the ground, or sits/stands while looking for food on the ground. Also includes active eating/ingesting/handling of food items.
**Aggression**		
Conspecific aggression	Event	Open-mouth threat: Individual opens his mouth for a while, directed at the receiver of aggression. Chin often pointed forward.<br>Chase: Individual runs after a conspecific for at least three seconds.<br>Lunge: Individual jumps a maximum of two body lengths towards a conspecific.<br>Stare: Individual looks intensely at a conspecific with a straight back and raised eyebrows in an attempt to threaten.<br>(any one of the above qualifies for conspecific aggression)
Predator aggression	Event	Stare: Individual looks intensely at the stressor with straight back and raised eyebrows for at least 3 seconds.<br>Vocalisation: Individual makes loud warning calls and barks at the stressor. The direction of the head always remains toward the stressor. During the process, the erection of body hair or piloerection can be seen.<br>(any one of the above qualifies for predator aggression)
**Self-directed behaviors**		
Autogroom	Event	Individual grooms oneself or closely inspects skin, nails, hands, toes or other body parts.
Scratch	Event	Individual uses fingers, hands, or foot to rake across own skin.
Freeze	Event	Individual maintains a tense body posture with no movement or vocalisations for at least 3 seconds.
Yawn	Event	Individual opens mouth wide and inhales intensely, which can be seen by the expansion of the chest.
**Conspecific affiliation**		
Groom and lip-smack towards conspecifics	State and Event	Groom: Individual touches and strokes another individual's fur gently with one or both hands, accompanied by periodic hand contact with their own mouth. Individual pays close attention to the recipient's fur (state).<br>Lip-smack: Individual opens and closes their lips rapidly, accompanied by a smacking sound, directed at a conspecific (event).<br>(any one of the above qualifies for conspecific affiliation)

Figure S2 – Categorization of individuals into different coping styles, related to STAR Methods. Individuals (n=30) were plotted and categorised into aggression-based, nonaggression-based and mixed coping styles based on scores obtained from the exploratory factor analysis. Individuals with low reactivities were also seen and represented. One individual (highlighted using a solid red dot), even though grouped into aggression-based, did not score higher than the population median (scores: aggression-based factor = 0.07, population median = 0.15). Subsequently, the individual was grouped as a low-reactant for analysis. Validation of results and downstream analyses were checked by including this individual as an aggression-based coper.

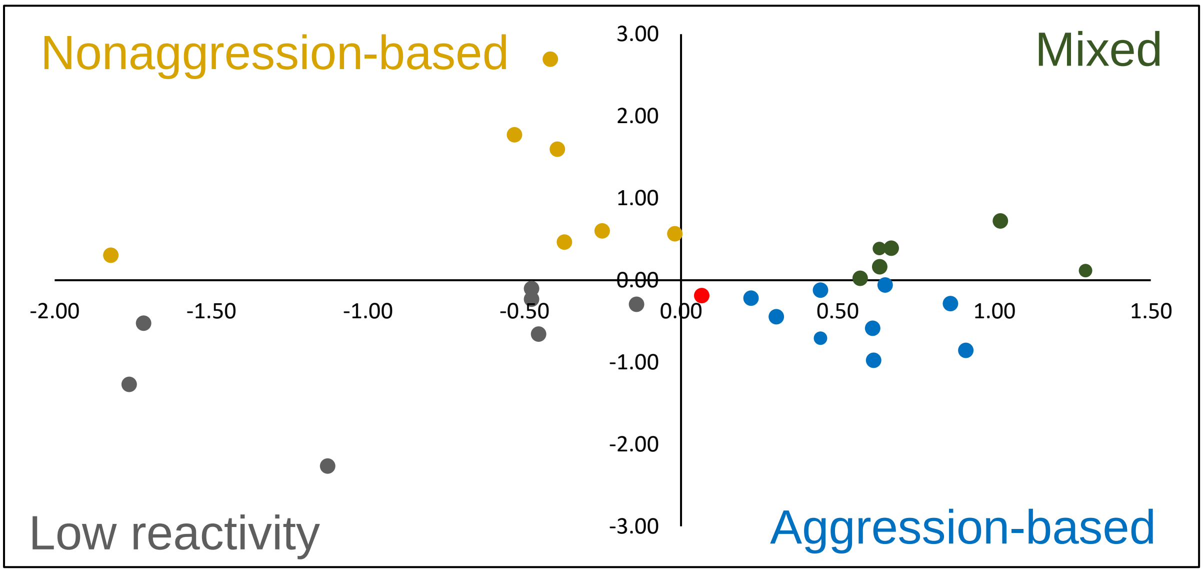

Table S2 – Effect of coping styles on nose temperature change, related to Figure 2. Summary of the linear mixed-effect model.

Model: Temperature change ~ coping style + time window + sex + (1|Group/id)

Fixed effects	Estimate	Std. error	df	t value	p-value
(Intercept)	-0.204	1.464	4.114	-0.140	0.895
Time window (10-20 min)	1.264	0.696	22.00	1.815	0.083
Time window (20-30 min)	1.315	0.696	22.00	1.889	0.072
Coping style (nonaggression)	-2.291	0.920	7.676	-2.490	0.038*
Sex	1.565	1.112	8.349	1.408	0.195

(Significance code: 0.01 ‘*’)

Figure S3 – Mid-nose temperatures of the aggression- and nonaggression-based copers at different time windows of the predator exposure experiments, related to Figure 2. The absolute average (± standard error) mid-nose temperatures of the aggression- and nonaggression-based copers (n=12) during predator exposure experiments.

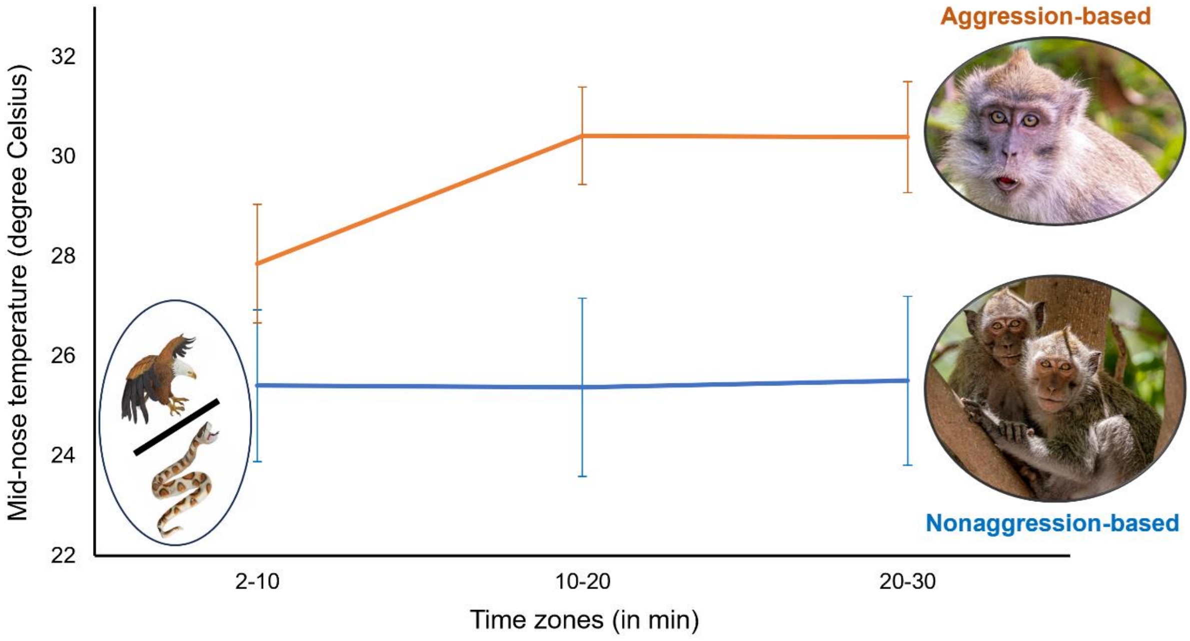

Table S3 – Effect of age and sex on activity-sociability trait, related to STAR Methods. Summary of the linear mixed-effect model and null-full model comparison results.

Model: Activity-Sociability ~ Age + Sex + (1|Group/id)

Fixed effects	Estimate	Std. error	z value	p-value
(Intercept)	0.178	0.274	0.648	0.516
Age	-0.096	0.035	-2.722	0.006 **
Sex (Male)	0.865	0.285	3.027	0.002 **

Comparison with null model: Likelihood ratio test - $\chi 2 = 18.17$, $p < 0.001$ ***
(Significance code: 0.01 ‘*’, 0.001 ‘**’, 0.000 ‘***’*)*

Table S4 – Effect of age and sex on exploration, related to STAR Methods. Summary of the linear mixed-effect model output and null-full model comparison results.

Model: Exploration ~ Age + Sex + (1|Group/id)

Fixed effects	Estimate	Std. error	z value	p-value
(Intercept)	0.435	0.271	1.602	0.109
Age	-0.131	0.036	-3.562	<0.001 ***
Sex (Male)	0.655	0.284	2.306	0.021 *

Comparison with null model: Likelihood ratio test - $\chi 2 = 21.001$, $p < 0.001$ ***
(Significance code: 0.01 ‘*’, 0.000 ‘***’*)*

Table S5 – Personality scores of the individuals, related to STAR Methods. Summary of the identities of the individuals, the groups they belonged to, and personality scores from the three personality dimensions.

Name	Activity-Sociability	Affiliation	Exploration
**Group 1**			
Alibi	-1.00	0.21	-0.25
Bowi	1.76	-0.87	-0.87
Dukki	-0.32	0.28	0.34
Impromptu	1.70	0.64	0.64
Mooi	-0.50	0.18	-0.59
Oui	1.47	-0.35	0.47
Tebbi	-0.37	2.54	1.14
Toffi	-0.71	-0.49	-0.22
Tutudetuu	-0.80	-2.32	1.89
Urbi-et-orbi	-0.83	0.46	-1.16
Walibi	-0.41	-0.28	-1.38
**Group 2**			
Castello	-0.74	1.49	-0.49
Elmo	0.41	-0.14	1.08
Emerald	0.28	-0.04	0.80
Etten	-0.19	-0.21	-0.55
Freggel	1.60	-0.60	2.17
Hacienda	-1.21	-1.10	-0.82
Lageveen	0.21	0.21	-0.26
Magelaen	-0.61	0.60	-1.13
Monopoly	2.62	-0.83	0.70
Nellie	-1.22	-1.43	1.22
Rizzo	-0.11	-0.11	-1.23
Rox	-0.26	1.18	-0.27
Saboteur	-0.71	0.24	-0.88
Sjors	1.48	-1.06	1.74
Soest	0.04	0.24	-0.96
Stas	-0.79	1.09	-0.65
Tamayo	-0.80	0.46	-0.47
**Group 3**			
Driel	0.30	0.78	-0.26
Horsten	0.12	-1.91	-0.81
Kluivers	-1.07	-0.22	1.42
Sollie	0.66	1.35	-0.35

Table S6 – Effect of personality on aggression-based coping, related to STAR Methods. Summary of the linear mixed-effect model output.

Model: Aggression-based coping score ~ Activity-sociability + Affiliation + Exploration + Age + Sex + (1|Group)

Fixed effects	Estimate	Std. error	t value	p-value
(Intercept)	0.644	0.184	3.499	0.006 **
Activity-sociability	-0.219	0.133	-1.639	0.135
Affiliation	-0.044	0.095	-0.460	0.656
Exploration	0.087	0.098	0.887	0.398
Age	-0.003	0.022	-0.141	0.890
Sex (Male)	0.077	0.227	0.339	0.742

(Significance code: 0.001 '**'*)*

Table S7 – Effect of personality on nonaggression-based coping, related to STAR Methods and Figure 3. Summary of the linear mixed-effect model output.

Model: Nonaggression-based coping score ~ Affiliation + Exploration + Age + Sex + (1|Group)

Fixed effects	Estimate	Std. error	t value	p-value
(Intercept)	3.080	0.337	9.119	0.011 *
Affiliation	-0.862	0.143	-6.003	0.026 *
Exploration	0.411	0.193	2.122	0.167
Age	-0.244	0.062	-3.943	0.058
Sex (Male)	-2.261	0.331	-6.818	0.020 *

(Significance code: 0.01 '*'*)*

Figure S4 - *COMT* Val/Met encoding polymorphism and personality traits, related to STAR Methods. The box plot shows the personality scores from the three traits and their association with *COMT* Val/Met genotype. Individual data points (n=26) are represented using solid dots. Boxes represent interquartile ranges, and whiskers represent the upper and lower limits of the data. The horizontal bars within the boxes represent the median values.

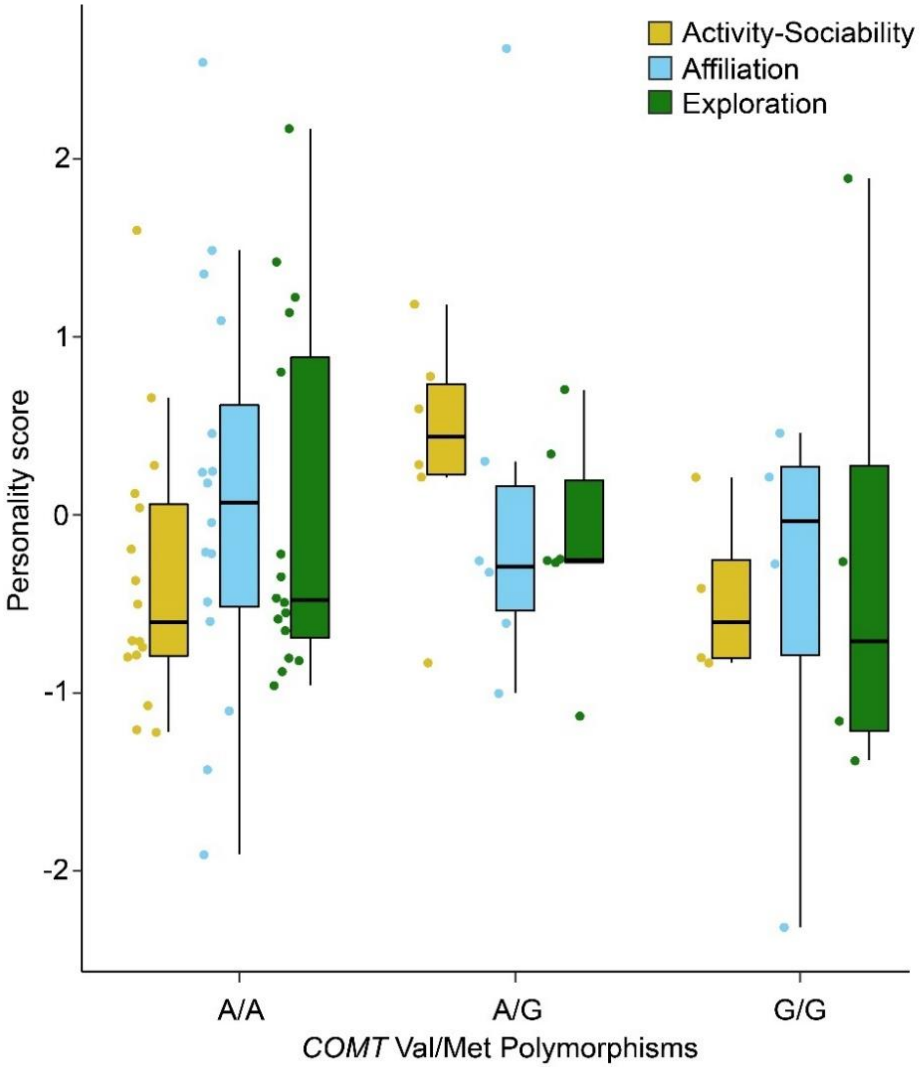

Table S8 – Details of the individuals in the study, related to STAR Methods. The identity, sex, relatedness, and date of birth of the participating individuals from the three groups are summarised. The placement of the macaques from left to right indicates mother-offspring relationships. For example, Bowi is the daughter of Walibi in Gr.1. Individuals who were not included in the *COMT* Val/Met polymorphism analyses are denoted with '*'.

Identity and sex of the individuals			Date of birth
**Group 1**			
Dukki ♀			13/10/2017
Mooi ♀			16/08/2017
Alibi ♀			18/10/2021
Toffi ♀			30/09/2017
Walibi ♀			10/09/2012
	Bowi ♀ *		18/10/2020
Urbi-et-orbi ♀			13/10/2014
	Tutudetuu ♂		11/11/2018
	Oui ♀ *		18/09/2020
Tebbi ♀			31/08/2017
	Impromptu ♂ *		29/01/2021
**Group 2**			
Rizzo ♂ *			24/10/2009
Castello ♀			14/01/2008
Saboteur ♀			17/08/2007
	Tamayo ♀		20/10/2017
Hacienda ♀			19/01/2008
Magelaen ♀			14/08/2007
	Soest ♀		11/05/2015
		Emerald ♀	09/04/2019
		Freggel ♂	09/04/2020
		Elmo ♂ *	15/02/2021
	Etten ♀		13/08/2016
	Rox ♀		18/04/2017
	Lageveen ♀		17/10/2018
	Monopoly ♂		08/06/2019
	Stas ♀		21/08/2014
		Nellie ♀	14/06/2019
		Sjors ♂ *	27/02/2021
**Group 3**			
Sollie ♂			12/12/2015
Horsten ♂			20/10/2017
Kluivers ♂			22/10/2017
Driel ♂			04/12/2017

Figure S5 – Predator models used in the study, related to STAR Methods. (a) Bird of prey resembling a hawk, (b) Snake resembling a python.

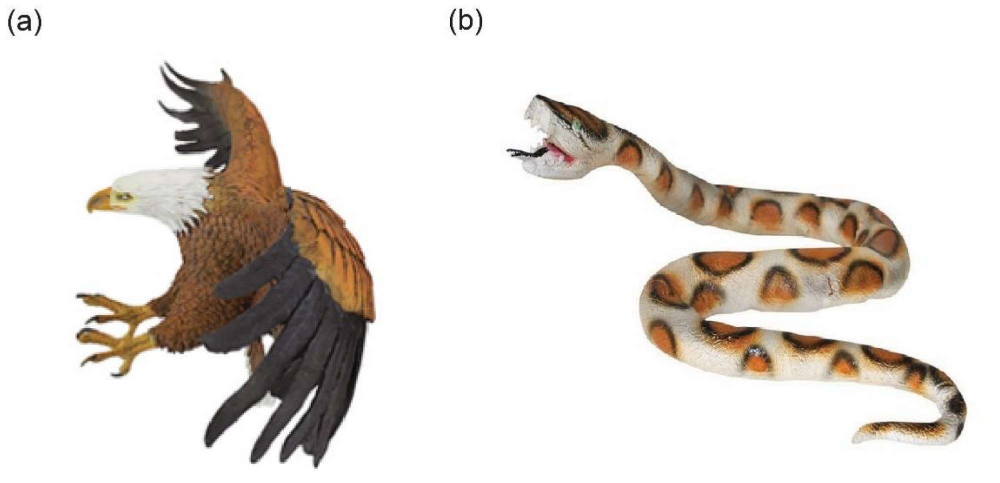

Figure S6 – Novelty experiments for personality assessment, related to STAR Methods. (a) Monkeys operating a food puzzle box to retrieve food rewards, (b) Upon rotating a food pipe puzzle, monkeys are obtaining rewards, (c) monkeys inspecting novel food rambutans and (d) dragon fruits, (e) monkeys holding egg containers and (d) massage rollers.

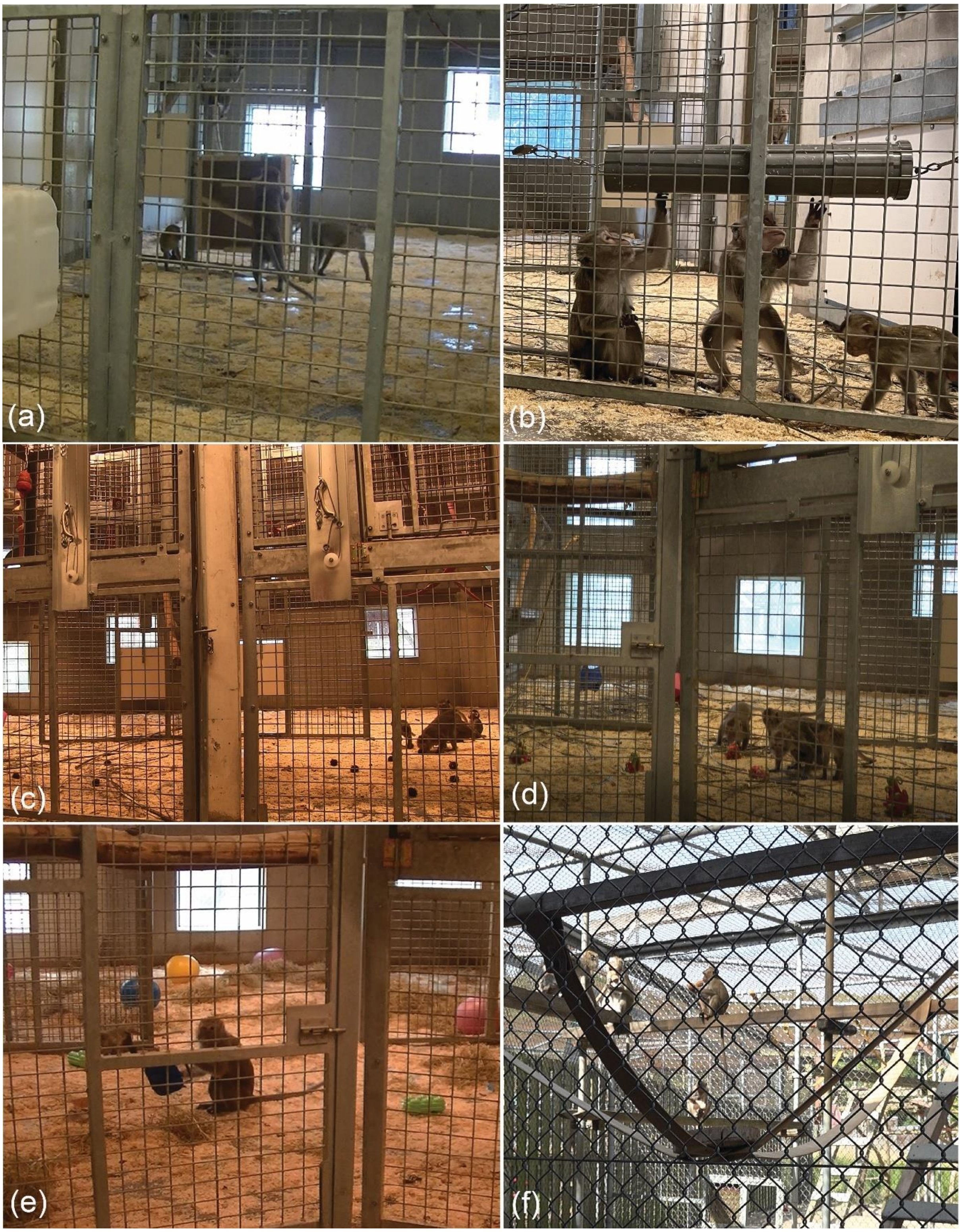

Table S9 – Intraclass correlation (ICC) results on coping-related behavioral variables, related to Figure 1 and STAR Methods. ICC values, 95% Confidence interval (CI), F- and p-values are summarised. Repeatable variables following a cutoff of 0.3 are presented in bold fonts. Behavioral variables highlighted in bold fonts were used in the final exploratory factor analysis.

Behavioral variable	ICC	95% CI; lower, upper	F-value	p-value
**Close ground**	**0.397**	0.049, 0.659	2.317	0.013
**Far**	**0.799**	0.620, 0.899	8.971	< 0.001
Locomotion	**0.548**	0.240, 0.756	3.429	< 0.001
Foraging	**0.319**	-0.040, 0.605	1.938	0.039
**Conspecific aggression**	**0.588**	0.295, 0.780	3.859	< 0.001
**Predator aggression**	**0.544**	0.234, 0.753	3.386	< 0.001
Autogroom	0.044	-0.315, 0.393	1.093	0.405
Scratch	0.042	-0.317, 0.391	1.088	0.410
Freeze	**0.547**	0.238, 0.755	3.415	< 0.001
Yawn	**0.669**	0.412, 0.827	5.053	< 0.001
**Conspecific affiliation**	**0.540**	0.229, 0.751	3.351	< 0.001

Figure S7 – Dominance hierarchies of the groups, related to STAR Methods. Based on submissive behaviors, the mean Elo-winning probabilities and 95% CI values of the monkeys (n=32) are plotted against their ordinal ranks. From top to bottom: Gr1. to Gr.3.

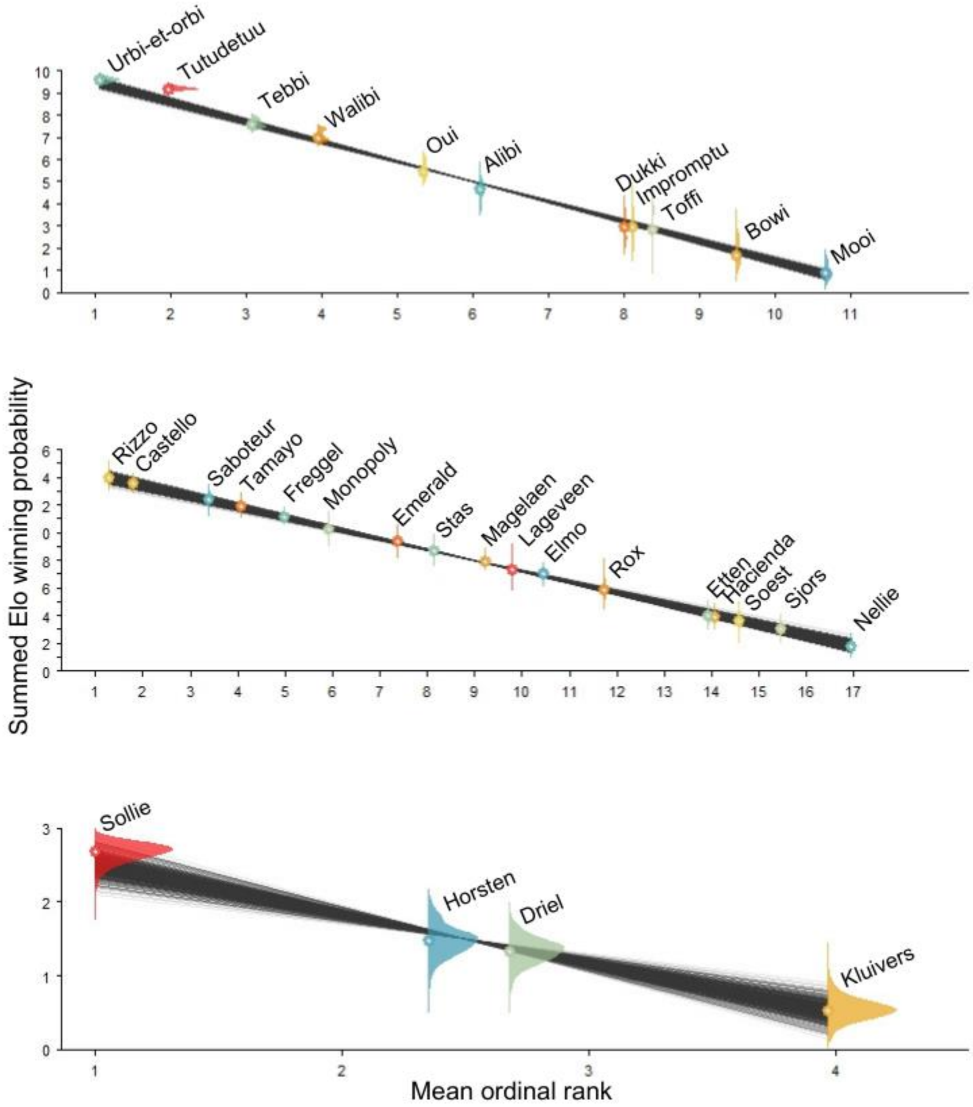

Supplement: Document S1. Figures S1–S7 and Tables S1–S9 [file mmc1.pdf]
